# Novel Inhibitors for MDM2-MDM4 E3 Ligase Potently Induce p53-Indepedent Apoptosis in Drug-Resistant Leukemic Cells

**DOI:** 10.3390/molecules30010186

**Published:** 2025-01-05

**Authors:** Rati Lama, Joseph M. Fose, Diana Martín, Inés G. Muñoz, Eunice S. Wang, Pamela J. Sung, Sherry R. Chemler, Xinjiang Wang

**Affiliations:** 1 Department of Pharmacology and Therapeutics, Roswell Park Comprehensive Cancer Center, Buffalo, NY 14263, USA; ratilama@hotmail.com (R.L.); pamela.sung@roswellpark.org (P.J.S.); 2 Department of Chemistry, University at Buffalo, State University of New York, Buffalo, NY 14260, USA; jmfose@buffalo.edu (J.M.F.); schemler@buffalo.edu (S.R.C.); 3 Structural Biology Programme, Spanish National Cancer Research Centre (CNIO), 28009 Madrid, Spain; diana.martin@inia.csic.es (D.M.); ines.g.munoz@gmail.com (I.G.M.); 4 Department of Medicine, Roswell Park Comprehensive Cancer Center, Buffalo, NY 14263, USA; eunice.wang@roswellpark.org

**Keywords:** MMRi36, MMRi36C, thiadiazole, MDM2-MDM4, E3 ligase, p53-independent, apoptosis, leukemia

## Abstract

MDM2 and MDM4 are major negative regulators of tumor suppressor p53. Beyond regulating p53, MDM2 possesses p53-independent activity in promoting cell cycle progression and tumorigenesis via its RING domain ubiquitin E3 ligase activity. MDM2 and MDM4 form heterodimer polyubiquitin E3 ligases via their RING domain interaction. Inhibitors disrupting p53 interaction with MDM2/MDM4 are in clinical trials in patients bearing wild-type p53 cancers. However, these inhibitors are not designed to work for p53-null/mutant cancer cells. Owing to the importance of the E3 ligase of MDM2 in its p53-independent oncogenic activity, inhibitors targeting the E3 ligase activity of MDM2-MDM4 are desirable for p53-mutant cancer cells. Here, we report the development of such inhibitors with pro-apoptotic activity in p53-null leukemic cells. Among analogues of MDM2-MDM4 E3 ligase inhibitors, we initially identified MMRi36 as a potent pro-apoptotic compound in p53-null leukemic cells with acquired drug resistance. MMRi36 acts as an activator of MDM2-MDM4 E3 ligase by stabilizing MDM2-MDM4 heterodimers and promotes MDM2/MDM4 degradation in cells. Interestingly, replacement of the sulfur in 1,3,4-thiadiazole MMRi36 with a carbon led to identification of pyrazole MMRi36C that dissociates the MDM2-MDM4 RING heterodimers, inhibits the E3 ligase activity of the complex, and induces p53 protein accumulation, but retains the p53-independent pro-apoptotic activity. A brief SAR study identified a fluorine derivative of MMRi36C with improved pro-apoptotic activity. This study discovered a novel class of compound that targets MDM2-MDM4 ubiquitin E3 ligase activity for apoptosis induction in p53-mutant cancer cells.

## 1. Introduction

**Figure 1:**
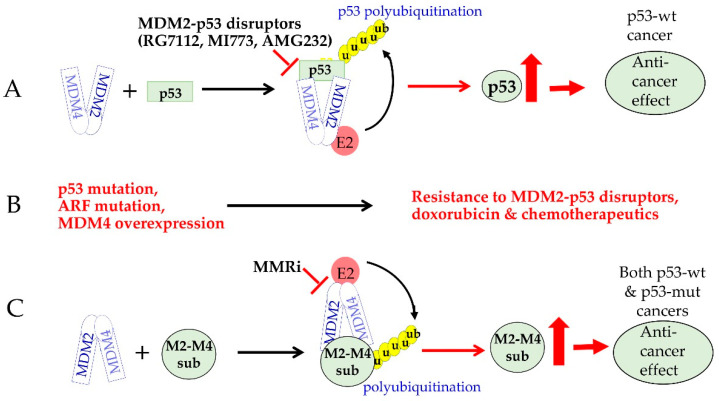
Advantages of targeting RING domain functions of MDM2-MDM4 in anti-cancer drug development. (**A**) Mechanisms of MDM2-p53 disruptor inhibitors and their anti-cancer effect in p53-wt cancers. (**B**) Components of the p53 pathway mutations or MDM4 overexpression confer resistance to MDM2-p53 disruptors and other chemotherapies in cancer. (**C**) Targeting the RING domain activity of MDM2-MDM4 by MMRi induces anti-cancer effect in both p53-wt and p53-mutant cancers.

Small-molecule inhibitors that target MDM2-p53 interaction have been pursued for decades for p53-based cancer therapies. These inhibitors bind to the MDM2′s p53-binding pockets and prevent MDM2-mediated inhibition of p53, leading to p53 protein accumulation and the unleashing of its potent tumor suppressor activity. Aided by the crystal structure of the MDM2-p53-binding pockets and computational chemistry, multiple potent and highly specific inhibitors with high binding affinity have been developed and advanced to clinical trials [1] (Figure 1A). Despite high hopes for these inhibitors being efficacious in patients bearing wild-type p53 cancers, they have two limitations. One is that these inhibitors will not work in p53-mutant cancers, the majority of which are high-staged cancers lacking effective treatment. Secondly, treatment with these inhibitors select p53 mutations and/or alterations in the components of the p53 pathway that confer resistance to these inhibitors [2,3,4] (Figure 1B). MDM4 (also known as MDMX), the MDM2 homologue, is also a resistance factor for MDM2-p53 disruptor inhibitors. MDM4 is often overexpressed by mechanisms including 1q gain [5] and MDM4 gene amplification [6,7]. Cancer cells expressing high levels of MDM4 have been shown to dampen the activities of MDM2-p53 disruptor Nutlin3a [8,9]. This is because MDM4 can compete with MDM2 for p53 binding and inhibits p53’s transactivation activity on its own. To circumvent the resistance by MDM4, inhibitors targeting both MDM2 and MDM4 have been reported [1].

In addition to p53 binding and inhibition of p53, MDM4 also binds to MDM2 via interaction of their RING domains [10]. The RING domains of both MDM2 and MDM4 are essential for p53 regulation in vivo [11,12,13]. This RING-RING interaction not only just stimulates MDM2’s intrinsic E3 ligase activity [14,15] but is also essential for MDM2’s polyubiquitin E3 ligase activity [16]. The polyubiquitin E3 ligase activity serves as the major mechanism for MDM2-MDM4 heterodimers in p53 regulation as well as for their p53-independent oncogenic activity. This was uncovered in our MDM2L466A mouse model expressing an intact RING domain that allows the formation of MDM2-MDM4 heterodimers without E3 ubiquitin ligase activity. The phenotypes of MDM2L466A mutant mice concluded that the E3 ligase activity of MDM2-MDM4 RING heterodimers was not only essential for p53 regulation in vivo but was also required for cell cycle progression independent of p53 [17]. Therefore, RING-RING interaction of MDM2-MDM4 is a valid new targeting interface for drug development to target MDM2/MDM4’s oncogenic activity mediated by p53-dependent and p53-independent mechanisms (Figure 1C).

We attempted to identify small-molecule compounds that inhibit the E3 ligase activity of RING-RING interaction of MDM2-MDM4 and obtained several primary hits designated as MMRi for MDM2-MDM4 RING inhibitors [18]. Instead of searching for highly specific RING domain inhibitors with limited cancer cell killing effect, we decided to identify leads with pro-apoptotic activity in p53-mutant cells among MMRi hit analogues and consequently we identified MMRi36 leading to development of MMRi36C5. In this report, we show that MMRi36 promotes cellular degradation of MDM2 and MDM4 and p53-independent apoptosis in leukemic cells including leukemic cells with acquired multi-drug resistance and identification of MMRi36C and 36C5 that act as MDM2-MDM4 E3 ligase inhibitors with improved pro-apoptotic activity in p53-null HL60VR leukemic cells with a multi-drug resistance phenotype.

## 2. Results and Discussion

### 2.1. MMRi36 Effectively Induced Apoptosis in Leukemic Cell Lines Regardless of p53 Status and Was More Potent than Daunorubicin in Apoptosis Induction

**Figure 2:**
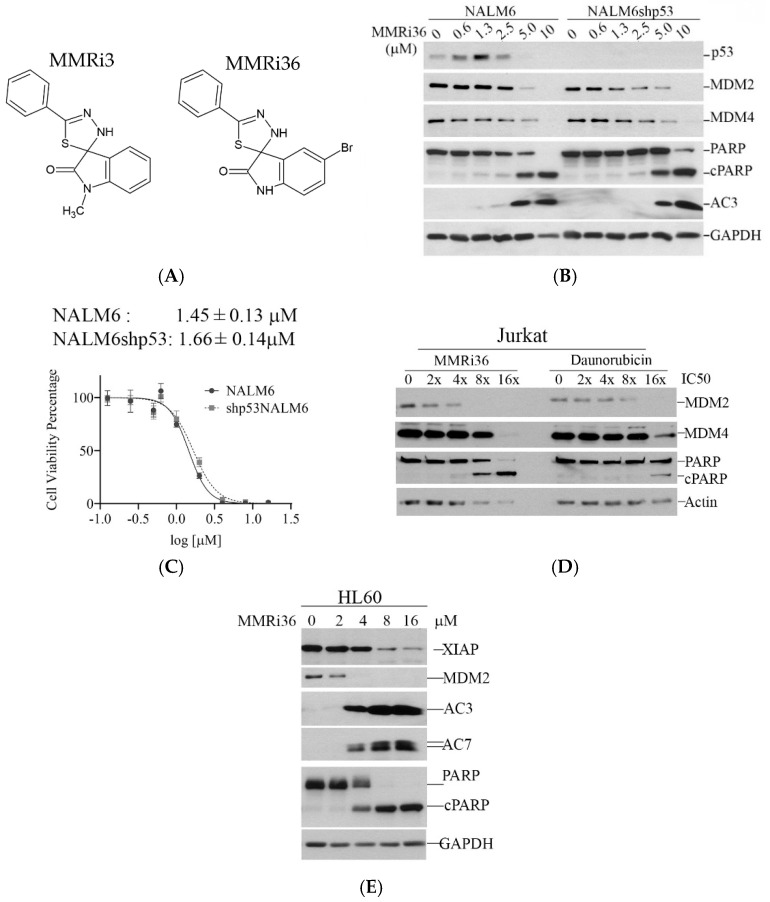
MMRi36 effectively induced apoptosis in leukemic cell lines regardless of p53 status and was more potent than daunorubicin in apoptosis induction. (**A**) Structure of MMRi3 and MMRi36. (**B**) NALM6 and NALM6shp53 lines were treated with indicated concentrations of MMRi36 for 24 h followed by Western blotting of p53, MDM2, MDM4, and apoptotic cleavage of PARP (cPARP) and activated caspase 3 (AC3). (**C**) Growth inhibition curves of NALM6 and shp53NALM6 cells in the presence of increasing doses of MMRi36 in 72 h proliferation assays. IC_50_s were calculated by median-effect CompuSyn software. (**D**) Comparison of apoptosis induction potency between MMRi36 and daunorubicin at equal effect doses (×IC_50_) for 24 h in Jurkat cells (MMRi36 IC_50_, 450 nM; daunorubicin, 5.78 nM). (**E**) MMRi36 effect on XIAP (Cell Signaling Technology, #2042S), and activation of caspase 3/7 and PARP cleavage in HL60 cells.

The primary hit MMRi3 is a thiadiazole identified in an enzyme-based HTS for inhibition toward the MDM2-MDM4 RING-domain-mediated E3 ligase reaction [18]. To find an E3 ligase inhibitor of the MDM2-MDM4 complex with p53-independent pro-apoptotic activity, we decided to use an apoptosis assay and p53-mutant cells to screen the available MMRi3 analogs from Hit2Lead library. This effort led to the identification of MMRi36. Interestingly, distinct from primary hit MMRi3 that inhibits the E3 ubiquitin ligase activity of MDM2-MDM4, MMRi36 stimulates the E3 ubiquitin ligase activity of MDM2-MDM4 in in vitro ubiquitination assays and promotes degradation of MDM2/MDM4 and p53 in cells [19]. The structures of MMRi3 and MMRi36 are shown in Figure 2A. To confirm that MMRi36-induced apoptosis is indeed p53-independent, we generated p53 knockdown leukemic cell line NALM6shp53 for testing. Our results from Western blotting analysis suggested that MMRi36 effectively induced an apoptosis response in both NALM6 and NALM6shp53 lines indicated by caspase 3 activation and PARP cleavage (AC3 and cPARP in Figure 2B). This apoptosis was associated with downregulation of MDM2, MDM4, and p53 proteins at the time when apoptosis was induced, consistent with the expected effect of MMRi36 as an activator of the MDM2-MDM4 E3 ligase complex. To confirm the effect of p53 status on MMRi36 sensitivity, we performed anti-proliferation assays with MMRi36 in these matched cell lines and found that the growth curves of the two cell lines in the presence of MMRi36 were nearly overlapped, with similar IC_50s_ (Figure 2C, 1.45, and 1.66 μM respectively). To ask whether MMRi36 is a more potent apoptosis inducer than daunorubicin, the first line treatment for leukemia, we treated Jurkat cells with different equal effect doses (×IC_50_) for 24 h and looked at cleaved PARP induction. Our results indicated that comparable level of cleaved PARP was induced by 4× IC_50_ dose of MMRi36 but 16× IC_50_ dose of daunorubicin (Figure 2D). Further comparison in more cell lines including leukemic CCRF-CEM, MV4-11, and p53-mutant lymphoma RAMOS cells reached the same conclusion that MMR36 induces significant apoptosis at 8× IC_50_, while daunorubicin at 16× IC_50_ failed to do so or only induced little apoptosis (S1). Using p53-null HL60 cells, we found that MMRi36-induced apoptotic PARP cleavage was accompanied with downregulation of MDM2, XIAP, and activation of caspase 3 and caspase 7 (Figure 2E). Taken together, these results suggest that MMRi36 has good pro-apoptotic activity in leukemic cells, regardless of p53 status.

### 2.2. Pro-Apoptotic Activity of MMRi36 Was Not Affected by Mechanisms for Daunorubicin Resistance in Leukemic Cells

**Figure 3:**
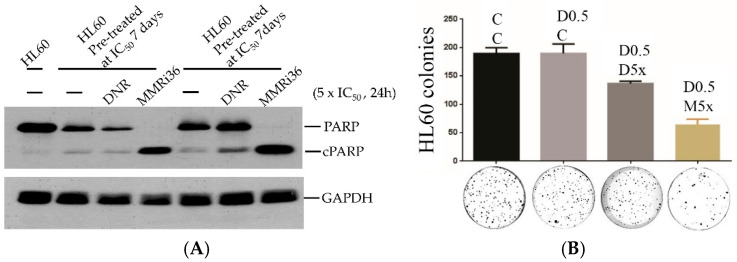
Pro-apoptotic activity of MMRi36 was not affected by daunorubicin resistance mechanisms. (**A**) HL60 cells pre-exposed to daunorubicin at IC_50_ (4.41 nM) or IC_75_ (12.77 nM) for one week. Then, the cells were replated and treated with 5× IC_50_ of either daunorubicin (22.05 nM) or MMRi36 (5.05 μM) for 24 h followed by WB for PARP cleavage. (**B**) HL60 cells pre-exposed to IC_50_ dose (D0.5) of daunorubicin as in (**A**), then were replated and treated with 5× IC_50_ of either daunorubicin (22.05 nM, D5x) or MMRi36 (M5x) for 12 h and then used in soft agar assays. Upper, histograms of the colony numbers of the indicated samples. Lower, images of colonies grown for 14 days in soft agar plates.

Drug resistance contributes to therapy failure of leukemia patients. Intrinsic drug resistance exists prior to drug treatment, while acquired drug resistance is developed during the drug treatment. To test whether MMRi36 is useful to induce apoptosis in leukemic cells with intrinsic resistance to daunorubicin, we treated HL60 cells at IC_50_ or IC_75_ dose for a week to enrich cells with intrinsic resistance to daunorubicin. We then challenged the cells with 5× IC_50_ of either daunorubicin or MMRi36 for 24 h. WB analysis indicated that daunorubicin challenge induced none or little apoptotic PARP cleavage in the conditioned cells by two pre-treatment conditions, indicating the viable cells of intrinsic daunorubicin resistance. In contrast, the challenge with MMRi36 induced complete apoptotic PARP cleavage in these cells (Figure 3A), suggesting that cells with intrinsic daunorubicin resistance remain sensitive to MMRi36-induced apoptosis. Furthermore, we plated the cells after 12 h challenge with either daunorubicin or MMRi36 in soft agar plates for colony formation assay. Our results showed that MMRi36 treatment significantly reduced the colony numbers compared to daunorubicin (Figure 3B). These results demonstrate that the pro-apoptotic activity of MMRi36 is not affected by daunorubicin resistance mechanisms.

**Figure 4:**
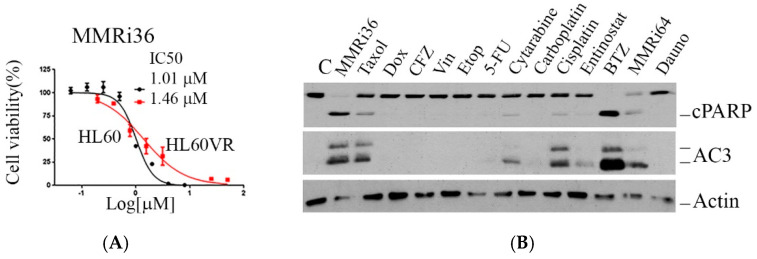

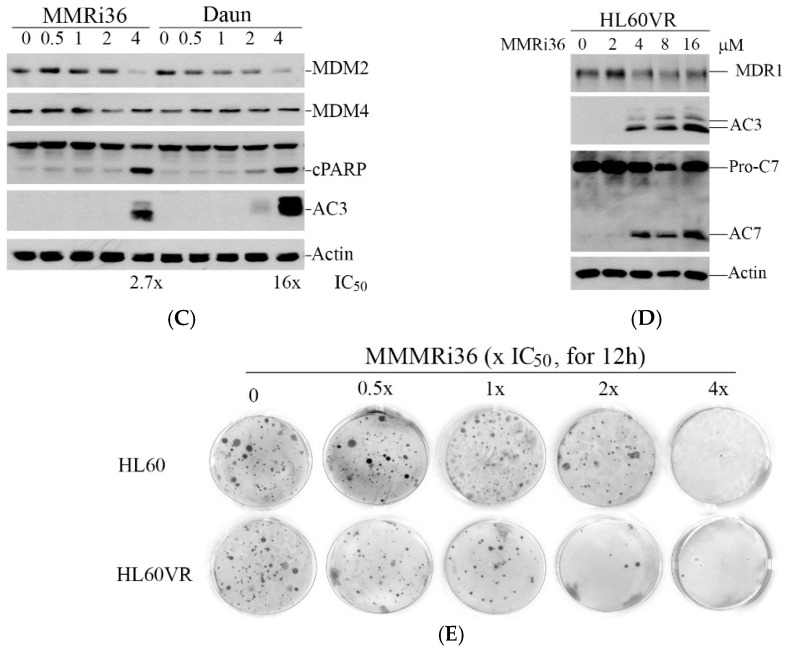
MMRi36 remains active in HL60VR cells with acquired drug resistance by potent apoptosis induction. (**A**) Growth curves and IC_50_s of parental HL60 and vincristine-resistant HL60 (HL60VR) in the presence of increasing concentrations of MMRi36. (**B**) Apoptotic response in HL60VR cells by different drugs. The cells were treated with vincristine at 333-fold IC_50_ in HL60 cells and other indicated drugs at 5× IC_50_ concentrations in HL60 cells for 24 h. Apoptosis induction was shown by PARP cleavage and activation of caspase 3 (AC3). (**C**) Comparison of apoptosis induction potency between MMRi36 and daunorubicin at equal concentrations for 24 h in HL60VR cells. (**D**) Effect of MMRi36 on MDR1 expression during apoptosis induction in HL60VR treated at the indicated concentrations for 24 h. (**E**) MMRi36 has comparable effects in 2D and 3D culture systems for both HL60 and HL60VR cells. Cells were treated with indicated concentration of MMRi36 for 12 h. Then, colonies were grown for 14 days. Colonies were stained with methylene blue solution and air-dried for imaging.

To know whether MMRi36 also has anti-leukemic activity in cells with acquired drug resistance, we tested it in the HL60-vincristine-resistant cell line (HL60VR), which was established by exposure of HL60 cells to increasing concentrations of vincristine over a long period of time [20]. HL60VR cells have a phenotype of resistance to apoptosis due to BCL-2 overexpression [21]. Compared to parental HL60 cells, the HL60VR cell line is 516-fold resistant to vincristine and 47-fold resistant to daunorubicin [22]. However, HL60VR and HL60 cells showed similar sensitivity to MMRi36 with IC_50_ of 1.01 and 1.46 μM (Figure 4A).

To compare MMRi36 with other chemotherapies for apoptosis induction in HL60VR cells, we treated HL60VR cells with 333x IC_50_ dose of vincristine and other drugs at 5× IC_50_ dose in HL60 cells for 24 h and performed WB analysis. Our results showed that MMRi36, taxol, cisplatin, bortezomib, and MMRi64 [18] could induce apoptosis in this multidrug-resistant cell line (Figure 4B). In HL60VR cells, MMRi36 was also a better apoptosis inducer than daunorubicin since 2.7× IC_50_ dose of MMRi36 (4 μM) induced similar PARP cleavage as 16× IC_50_ dose of daunorubicin (4 μM) (Figure 4C), and both caspase 3 and caspase 7 were activated by MMRi36 at concentrations higher than 4 μM (Figure 4D). The soft agar assay results indicated that 12 h exposure to MMRi36 nearly eliminated colony-forming tumor-initiating cells (Figure 4E). Collectively, these data demonstrate that MMRi36’s pro-apoptotic activity was not abrogated by leukemic cells with the acquired drug resistant phenotype.

### 2.3. Replacement of Sulfur with Carbon in MMRi36 Converted the E3 Ligase Activator to an E3 Ligase Inhibitor MMRi36C

**Figure 5:**
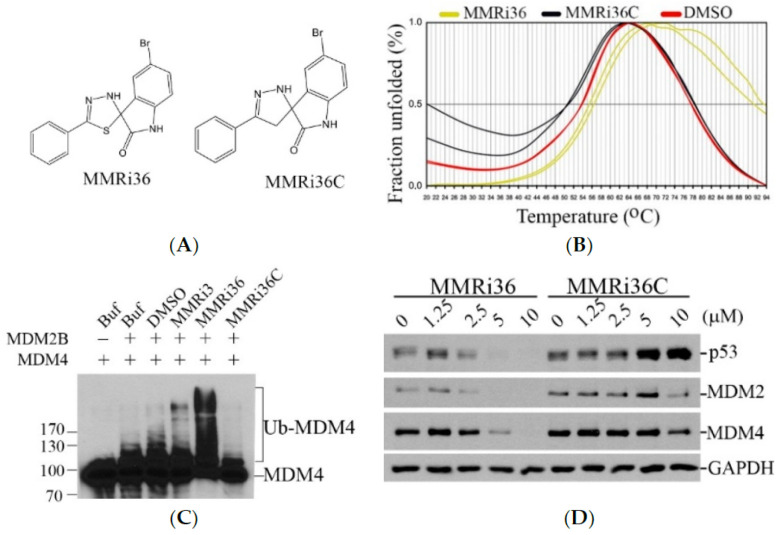
MMRi36C dissociated RING-RING interaction and acted as an inhibitor of MDM2 E3 ligase activity. (**A**) Chemical structure of MMRi36 and MMRi36C. (**B**) Thermofluor results of MMRi36 vs. MMRi36C using purified MDM2-MDM4-RING domain protein heterodimers. (**C**) In vitro ubiquitin E3 ligase assay using recombinant MDM4 and MDM2B protein in the presence of 10 μM indicated compounds. (**D**) MMRi36C was a more potent inducer of p53 protein accumulation than MMRi36. WB analysis of p53 in NALM6 cells treated with the indicated compound at the indicated concentrations for 24 h. GAPDH served as the loading control.

The chemical structure of MMRi36 contains a five-membered heterocyclic ring, 1,3,4-thiadazole, which plays an important role in cytotoxicity of thiadiazole-containing compounds as well as in their hepatotoxicity [23,24,25]. However, a pyrazole scaffold is preferred to thiadiazole since it is more chemically stable, and many FDA-approved drugs contain the pyrazole scaffold, including Avapritinib, Asciminib, Crizotinib, Encorafenib, and many others [26,27]. Therefore, we converted the 1,3,4-thiadazole core with a pyrazole scaffold by replacing the sulfur with a carbon atom in the ring structure, resulting in MMRi36C (Figure 5A). To understand how this change affects the effect of the compounds on RING-RING heterodimers of MDM2 and MDM4, we performed thermofluor (TF) assays with the recombinant RING heterodimers purified from *E. coli*. Our TF results showed that MMRi36 stabilized the RING heterodimers by increasing the *Tm* from 53.8 °C to 56.4 °C. In contrast, MMRi36C decreased *Tm* from 53.8 °C to 50.8 °C (Figure 5B), indicating that MMRi36C destabilizes RING-RING heterodimers in vitro. These Tm results explain why MMRi36 has an activating effect on the E3 ligase activity of MDM2-MDM4 and predicts that MMRi36C would have an inhibitory effect on the E3 ligase activity of MDM2-MDM4. Indeed, our in vitro MDM2-MDM4 E3 ligase assay results showed that MMRi36 stimulated while MMRi36C inhibited the E3 ligase activity using the production of ubiquitinated MDM4 as a readout (Figure 5C). The differential effects of MMRi36 and MMRi36C on the E3 ligase activity of MDM2-MDM4 in vitro is expected to result in a differential effect on p53 protein accumulation in cells. WB analysis indicated that MMRi36C is a more potent inducer of p53 accumulation than MMRi36 in NALM6 cells (Figure 5D). Taken together, replacement of thiadiazole with pyrazole scaffold led to a scaffold-hop of MMRi36 to MMRi36C with a change of their mechanism of action on their initial target of the MDM2-MDM4 E3 ligase, i.e., from an MDM2-MDM4 E3 ligase activator to an MDM2-MDM4 E3 ligase inhibitor with improved activity over MMRi3.

### 2.4. MMRi36C Retained Capability of Inducing p53-Independent Apoptosis, Downregulating MDM2/MDM4 and Inhibiting Growth of Drug-Resistant HL60VR Cells

**Figure 6:**
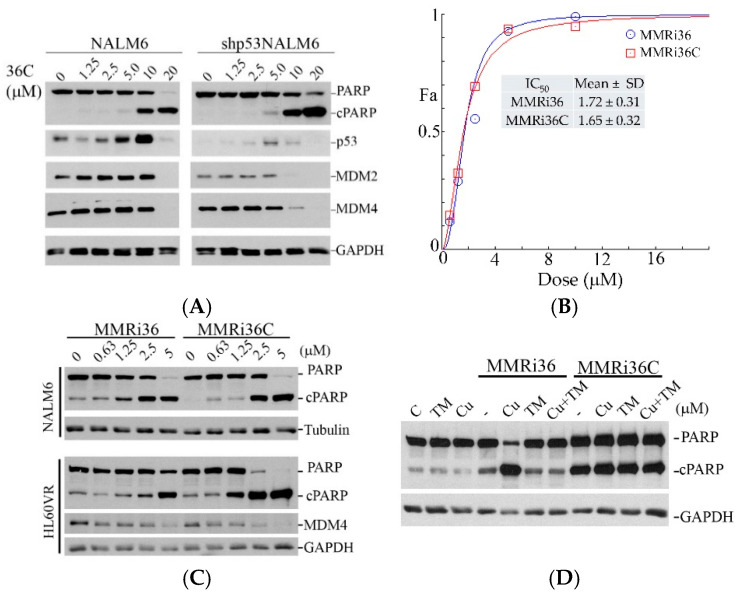
MMRi36C induced p53-independent apoptosis in leukemic cells. (**A**) WB analysis of p53, MDM2, MDM4, and cleaved PARP (cPARP) in NALM6 and shp53NALM6 cells after treatment with MMRi36C at the indicated concentrations for 24 h. (**B**) Growth inhibition curves of HL60VR cells treated with MMRi36 or MMRi36C. Date are representative of three independent experiments analyzed by the Chou–Talalay method with CompuSyn software. IC_50s_ and standard deviation (SD) are shown. Fa, affected fraction. (**C**) WB analysis of cleaved PARP (cPARP) in NALM6 and HL60VR cells after treatment with MMRi36 and MMRi36C at the indicated concentrations for 24 h. (**D**) The pro-apoptotic activity of MMRi36 but not MMRi36C is affected by the copper ion. WB analysis of PARP and GAPDH in HL60VR cells treated with MMRi36 or MMRi36C with or without 2 μM copper (II) sulphate or copper (II) chelator TM for 24 h. GAPDH served as the loading control.

The dramatic change of the mechanisms of action between MMRi36C and MMRi36 toward MDM2-MDM4 E3 ligase activity raised a question as to whether MMRi36C still retains its p53-independent pro-apoptotic activity. To answer this question, we performed WB analysis of NALM6 and shp53NALM6 cells treated with MMRi36C. Our results showed that a significant level of cleaved PARP was induced by MMRi36C at concentrations above 10 μM in both NALM6 and shp53NALM6 cells (Figure 6A). Of note, significant downregulation of MDM2 and MDM4 proteins was observed only in shp53NALM6 cells but not in NALM6 cells, possibly due to compensation by p53-mediated upregulation of MDM2 and stabilization of MDM4 in p53-wt NALM cells. Nevertheless, these results confirmed that MMRi36C retained the p53-indepndent pro-apoptotic activity in leukemic cells. We then tested MMRi36C together with MMRi36 in drug-resistant HL60VR cells and obtained IC_50s_ of 1.72 μM and 1.62 μM, respectively, for MMRi36 and MMRi36C (Figure 6B). Side-by-side comparison of MMRi36 with MMRi36C in cleaved PARP induction suggested that there was no significant difference between the two compounds in p53-wt NALM6 cells (Figure 6C, upper panel). Interestingly, MMRi36C appeared more effective in PARP cleavage induction in drug-resistant HL60VR cells (Figure 6C, lower panel). MMRi36 belongs to 1,3,4-thiadazole, some of which are metal ion chelators [28]. We tested the effect of ferrous, copper (II), and Zn on the pro-apoptotic activity of MMRi36 in cancer cells and found that copper (II) significantly increased the MMRi36-induced PARP cleavage and p53 accumulation in leukemic cells. We then tested the copper (II) effect on MMRi36 and MMRi36C side-by-side in PARP cleavage induction in HL60VR cells. We treated HL60VR cells with 2 μM MMRi36 or MMRi36C in the presence of 2 μM Cu (II) with or without including 2 μM copper chelator tetrathiomolybdate (TM). Our results showed that the MMRi36-induced PARP cleavage was significantly increased by Cu (II), an effect completely canceled by the presence of TM, indicating a strong effect of Cu (II) on MMRi36’s pro-apoptotic activity. In contrast, the MMRi36C-induced PARP cleavage was not affected by copper (II) or TM or both (Figure 6D). These results suggest that unlike MMRi36, MMRi36C induces apoptosis by mechanisms not involving metal chelation in leukemic cells.

### 2.5. Structural Requirement of MMRi36C in p53-Independent Apoptosis Induction

**Figure 7:**
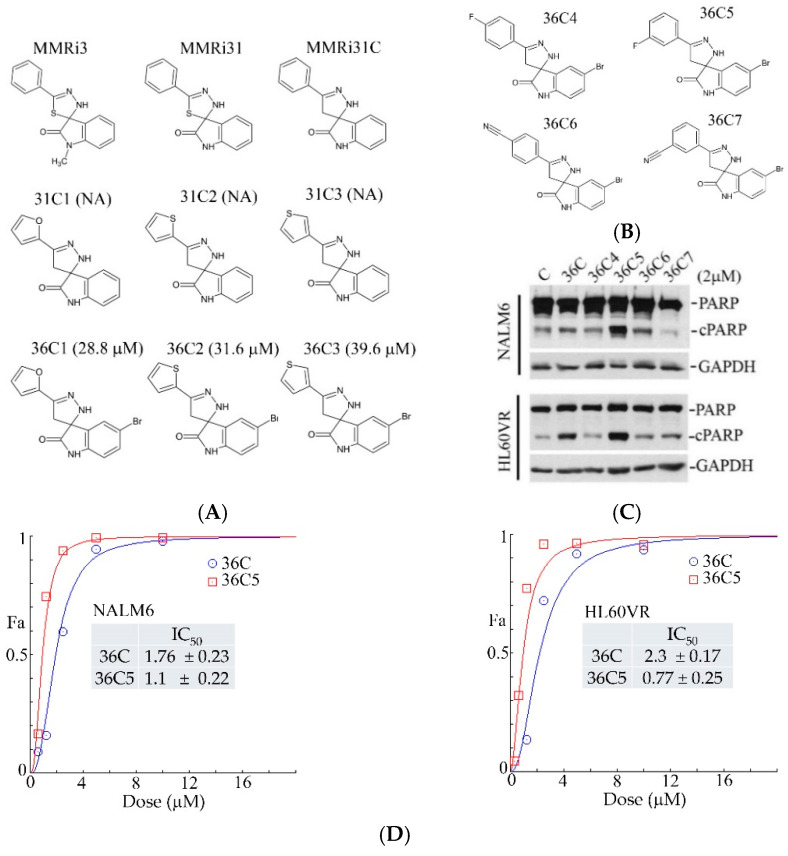

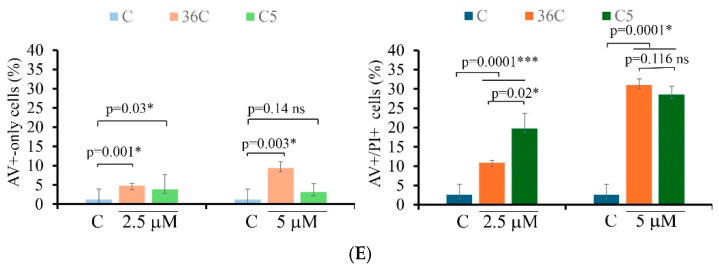
Structure–activity relationship study of MMRi36C. (**A**) Structures of MMRi3 derivatives with activities in brackets. NA, no activity. (**B**) Structures of MMRi36C derivatives. (**C**) WB for apoptotic PARP cleavage (cPARP) in NALM6 and HL60VR cells treated at 2 μM of each compound for 24 h. GAPDH served as the loading control. (**D**) Growth inhibition curves of MMRi36C and 36C5 in NALM6 (upper) and HL60VR (lower) cells. Data are representative of three independent experiments analyzed by the Chou–Talalay method with CompuSyn software. IC_50s_ and standard deviation (SD) are shown in the graphs. Fa, affected fraction. (**E**) Quantification of apoptosis induced by MMRi36C and 36C5 in HL60VR cells. Cells were treated by MMRi36C and 36C5 at 2.5 and 5 μM for 24 h followed by annexin-V-Alexa-488, PI, and DAPI staining. The annexin-V-positive apoptotic cells were quantified by a Cytation 5 imaging system. The fractions (%) of annexin-V-positive apoptotic cells (left panel) and annexin-V-positive/PI-positive (late stage apoptotic) cells (right panel) are shown. *p* values of unpaired Student’s *t* tests are shown: significant difference (*) or extremely significant (***) or no significant difference (ns).

A brief structure–activity relationship (SAR) study on MMRi36C was performed where the two arene rings were varied. We used MMRi31C and MMRi36C as starting structures and linked furan or thiophene to the pyrazole from different positions in six new compounds (Figure 7A). Our activity assay results showed that the three MRi31C derivatives totally lost activity while the three MMRi36C derivatives had significantly reduced activity with increased IC_50s_ of >30 μM, suggesting that the benzene ring and bromine are required for the activity and hydrophobic interaction might be involved in drug target binding.

Then, we designed and synthesized four new derivatives with bromine and benzene ring modified with either a fluorine or a cyanide group at different positions (Figure 7B). An initial activity assay showed that the two cyanide derivatives and 4-fluorophenyl derivative significantly lost activity in both NALM6 and HL60VR cells. However, the 3-fluorophenyl derivative 36C5 showed good activity. WB blotting for apoptotic PARP cleavage showed that 36C5 induced more robust PARP cleavage in both p53-wt NALM6 cells and drug-resistant p53-null HL60VR cells treated with 2 μM of C-serial compounds for 24 h (Figure 7C). We then compared 36C5 with MMRi36C for their anti-proliferation activities in NALM6 and HL60VR cells. The results showed that 36C5 was more active than MMRi36C in both cell lines with decreased IC_50_ compared to those of MMRi36C (Figure 7D). Then, we quantified the apoptotic fractions of HL60VR cells treated with MMRi36C and 36C5 for 24 h. At a lower concentration of 2.5 μM, both MMRi36C and 36C5 induced significantly higher apoptotic cells in early-stage apoptotic fraction (annexin-V-positive-only, Figure 7E left) and late-stage apoptotic fraction (annexin-V-positive/PI-positive, Figure 7E, right) cells compared to the non-treated control cells. 36C5 at 2.5 μM induced significantly higher late-stage apoptotic fractions than 2.5 μM MMRi36C (19.8 ± 4.1% for 36C5 versus 10.9 ± 0.8% for MMRi36C, *p* = 0.02). At higher concentrations of 5 μM, both compounds induced a significant increase in late-stage apoptotic fractions (31 ± 1.9% for MMRi36C and 29 ± 1% for 36C5) (Figure 7E right). The apoptotic cells induced by 5 μM 36C5 only had ~3% in early-stage apoptosis at 24 h, and most of them were in late-stage apoptosis. In contrast, the apoptotic cells induced with 5 μM MMRi36C still had ~9% in early-stage apoptosis (Figure 7E left). Since 5 μM 36C5-treated samples had much more apoptotic bodies than those treated with 5 μM MMRi36C, no significant difference was observed in the late-stage apoptotic fractions of 5 μM MMRi36C and 5 μM 36C5 (Figure 7E right, *p* = 0.116), which might have been due to the fact that the two compounds at 5 μM both reached their maximal effect as determined by their growth inhibition curves (Figure 7D). Collectively, these data suggest that 36C5 is a better apoptosis inducer than MMRi36C.

In summary, this study started from the identification of MMRi36 that acts as an activator of MDM2-MDM4 E3 ligase with potent p53-independent pro-apoptotic activity to the identification of MMRi36C by scaffold–hopping as an inhibitor of MDM2-MDM4 E3 ligase and identification of 3-fluorophenyl derivative 36C5 with improved activity. The mechanisms of action for these compounds have yet to be elucidated, although they act on the RING-RING heterodimers of MDM2-MDM4 *in vitro* and downregulate MDM4 in cells. Importantly, MMRi36, MMRi36C, and 36C5 all potently induce apoptosis in multi-drug-resistant p53-null HL60VR cells, a unique feature that distinguishes them from conventional MDM2/MDM4-p53 disruptor inhibitors in clinical trials. Therefore, this study identified the first-in-class small-molecule inhibitor of the oncogenic MDM2-MDM4 E3 ligase complex for p53-independent apoptosis induction, and 36C5 can serve as a starting structure for future comprehensive SAR research aiming to identify clinically useful anticancer therapeutics. Although XIAP protein downregulation was observed in MMRi36-induced apoptosis, whether this significantly contributes to apoptosis induced by MMRi36C and 36C5 is not known. Identification of all possible cellular targets of 36C5 and their assessment in apoptosis will help understand the mechanisms of action as well as development of 36C5 derivatives with improved drug-like properties.

## 3. Materials and Methods

### 3.1. Representative Chemistry Methods

The pyrazole derivatives investigated in this study (Figure 7) were synthesized and characterized in house, analogous to the reported methods [29,30,31,32,33]. They were dissolved in DMSO as 10 mM stock solutions for use in cell treatment. MMRi36 and MMRi31 were synthesized as previously reported [29]. MMRi31C was synthesized as previously reported [31]. MMRi3 was synthesized as previously reported [32]. Analogs 31C2 and 31C3 were synthesized as previously reported [33]. Novel derivatives 31C1-3 and 36C1-7 were synthesized via a three-step condensation of the respective acyl arene with the respective isatin and hydrazine in analogy to 31C2 and 31C3 [33]. The purity of each analog compound that was subjected to cellular and biochemical assays was assessed by each compound’s 1H NMR spectra, acquired at 400 MHz [34]. Details of chemical synthesis and compound characterization by mass spectrometry are provided in the Supplemental Information (S2).

### 3.2. Biological Assays and Methods

#### 3.2.1. Cell Culture

NALM6 and shP53NALM6 and HL60VR leukemic cell lines were cultured in RPMI-1640 medium supplemented with 10% fetal bovine serum, 50 U/mL penicillin, and 50 μg/mL streptomycin. The shP53NALM6 cell line was established using pLKO.1-p53 (purchased from Addgene) (Plasmid #19119) [35] followed by puromycin selection at 1 μg/mL for 2 days then clonal expansion in puromycin-free medium. Melanoma cell line A375 was cultured in DMEM-10% fetal bovine serum, 50 U/mL penicillin, and 50 μg/mL streptomycin.

#### 3.2.2. Western Blotting and In Vitro Ubiquitination

The Western blotting procedure and antibodies for the target proteins were described previously [22]. In vitro assays for ubiquitination by MDM2B-MDM4 were performed as described previously with minor modification [16]. Briefly, reactions were carried out at 30 °C for 1h in a volume of 20 μL reaction in the presence of different concentrations of compounds or vehicle solvent DMSO, followed by WB of p53 with DO-1, MDM2 with rabbit anti-MDM2 (MDM2 (D1V2Z) Rabbit mAb #86934, Cell Signaling Technology, Danvers, MA, USA), or MDM4 with a rabbit anti-MDM4 antibody (Proteintech, Cat no: 17914-1-AP, Rosemont, IL, USA). Apoptotic response to compounds was measured by Western blotting using specific antibodies from Cell Signaling Technology for activated caspase 3 (Cleaved Caspase-3 (Asp175) (5A1E) Rabbit mAb, #9664) and PARP (PARP Antibody #9542). XIAP was detected with rabbit antibody from Cell Signaling Technology (#2042S) or purified mouse antibody from BD (#610763).

#### 3.2.3. IC_50_ Measurement and Analysis

The procedure was described previously [22]. Briefly, cells at 10,000/well were plated in 96-well plates at 100 μL/well, and compounds of different concentrations were added to each well at 100 μL/well. After 70 h treatment, 40 μL of 6×resazurin stock solution was added to each well, followed by 2 h development of fluorescent metabolite by viable cells before reading OD600 in a BioTek Synergy 2 Microplate Reader (Agilent, Santa Clara, CA, USA). The IC_50_ values and some growth inhibition curves were obtained by the Chou-Median-Effect Equation using CompuSyn software [36], and some dose-effect curves were obtained by GraphPad Prism 8 using affected fractions of compound-treated wells normalized against no-drug control wells with a non-liner regression model.

#### 3.2.4. Quantification of Apoptotic Cells

Annexin-V staining kit (ThermoFisher Scientific, cat# V13245, Waltham, MA, USA) and BioTek Cytation 5 Cell Imaging Multimode Reader (Agilent, Santa Clara, CA, USA) were used for the quantification of apoptotic fractions in treated cells. Cells were treated with compounds for 24 h. Then, 0.3 × 10^6^ cells were withdrawn from the flasks and washed two times with 1 mL PBS in Eppendorf tubes. Then, the cells were resuspended in 300 μL of 1× annexin-V-binding buffer and incubated with 10 μL annexin-V-Alexa Fluor-488 (green) and 10 μL of propidium iodide (red) at RT for 15 min in a dark box. After washing the cells two times with 1 mL of 1 × annexin-V-binding buffer, the cells were resuspended in 400 μL of 1 × annexin-V-binding buffer and transferred to a 96-well black plate (Greiner, cat# 655090) at 100 μL per well. After mixing the 100 μL cells with 10 μL NucBlue™ Live ReadyProbes™ Reagent (Hoechst 33342 new formulation, Invitrogen, R37605, Waltham, MA, USA) for nuclear stain, the plate was mounted on a Cytation 5 system for capturing blue (cell count), green (annexin-V), and red (PI) fluorescence images. Apoptotic fractions were obtained as annexin-V-positive cell fractions (early-stage apoptotic) and annexin-V-positive/PI-positive (late-stage apoptotic) fractions.

## 4. Patents

Provisional patent: “Inducing Apoptosis” U.S. Patent Application No. 63/656,481. submitted.

## Data Availability

All data are included in the article and/or Supplementary Materials.

## Supplementary Materials

The following supporting information can be downloaded at https://www.mdpi.com/article/10.3390/molecules30010186/s1. Figure S1: Comparison of MMRi36 with daunorubicin in apoptosis induction in multiple cell lines; S2: Synthesis supporting information.
