# Supplementary material for: Novel Inhibitors for MDM2-MDM4 E3 Ligase Potently Induce p53-Indepedent Apoptosis in Drug-Resistant Leukemic Cells
Source: Molecules. 2025 Jan 5;30(1):186. doi: 10.3390/molecules30010186 (PMC11722259; doi:10.3390/molecules30010186)
Supplement: Supplementary file 1 [file molecules-30-00186-s001.zip › molecules-3384227-supplementary.pdf]

S1. Comparison of MMRi36 with daunorubicin in apoptosis induction in multiple cell lines.

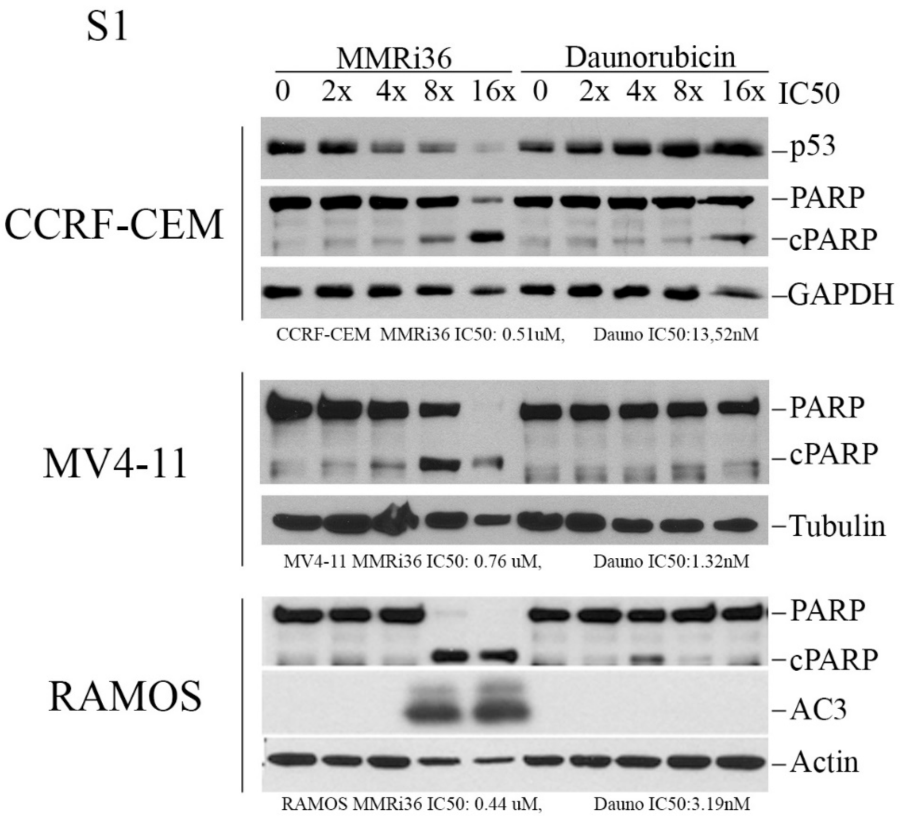

S1. Comparison of MMRi36 with daunorubicin in apoptosis induction in multiple cell lines. The indicated cell lines were treated with MMRi36 and daunorubicin at equal effect doses (× IC50) for 24h followed by WB analysis of apoptotic PARP cleavage (cPARP) or activated caspase 3 (AC3).

Novel inhibitors for MDM2-MDM4 E3 ligase potently induce p53-independent apoptosis in drug-resistant leukemic cells

Rati Lama[1], Joseph M. Fose[2], Ines G. Munoz[3], Eunice S. Wang[4], Pamela J. Sung[1,4], Sherry R. Chemler[2] and Xinjiang Wang[1]*

1. Department of Pharmacology and Therapeutics, Roswell Park Comprehensive Cancer Center, Buffalo, NY 14263, USA
2. Department of Chemistry, University at Buffalo, Buffalo, NY 14260, USA
3. Structural Biology Programme, Spanish National Cancer Research Center (NCIO), Madrid, Spain
4. Department of Medicine, Roswell Park Comprehensive Cancer Center, Buffalo, NY 14263, USA

*Correspondence: Xinjiang.wang@roswellpark.org

Synthesis Supporting Information (SRC, schemler@buffalo.edu)

General experimental information:
All substrates and solvents were reagent grade as provided by the chemical vendor. All nuclear magnetic resonance (NMR) data were collected using a Varian or Bruker 300 or 400 MHz spectrometers. All chemical shifts are reported in ppm and were referenced to residual solvent peaks (^{1}H NMR: DMSO-d_6 δ = 2.50 ppm, ^{13}C NMR: DMSO-d_6 δ = 39.5 ppm). Multiplicities are abbreviated as follows: singlet (s), doublet (d), triplet (t), quartet (q), quint (quintet), multiplet (m). Coupling constants are reported in Hertz (Hz). Infrared spectra were recorded using a Perkin Elmer Spectrum Two spectrometer using the attenuated total reflectance attachment (ATR). Wavenumbers in inverse centimeters (cm^{-1}) are reported for characteristic peaks. High resolution mass spectra were obtained at the University at Buffalo's mass spectrometry facility on a ThermoFinnigan MAT95XL high resolution magnetic sector mass spectrometer and Bruker Daltonics SolariX12 Tesla FTICR mass spectrometer. All EI and ESI data are reported as either $[M]^+$, $[M + 1]^+$, or $[M + Na]^+$. Melting points were measured with a Mel-Temp melting point apparatus and are uncorrected.

MMRi36 and MMRi31 were synthesized as previously reported.[1] MMRi36C was synthesized as previously reported.[2] MMRi31C was synthesized as previously reported.[3] MMRi3 was synthesized as previously reported.[4] Analogs 31C2 and 31C3 were synthesized as previously reported.[5] Analogs 31C1 and 36C1-7 were synthesized following the method reported for 31C2 and 31C3.[5] The purity of each analog compound that was subjected to cellular and biochemical assays was assessed by each compound's ^{1}H NMR spectra, acquired at 400 MHz.[6]

Representative procedure A:

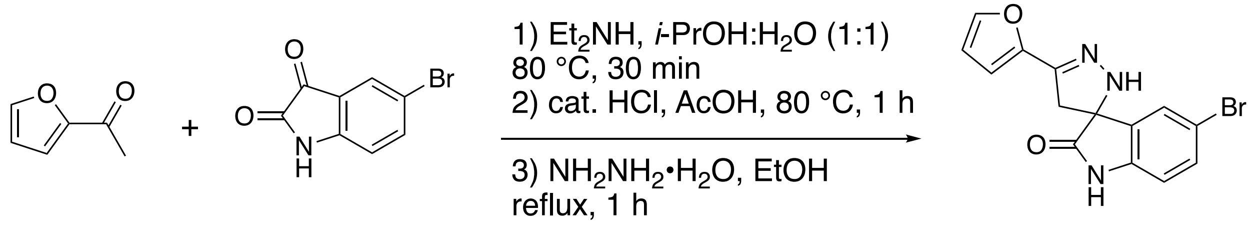

36C1

5-Bromo-5'-(furan-2-yl)-2',4'-dihydrospiro[indoline-3,3'-pyrazol]-2-one (36C1**)**

Following the procedure described by Youssef,[5] 2-acetylfuran (1 mmol) was converted to 36C1. 2-Acetylfuran (100 μL, 1.00 mmol) and 5-bromoisatin (228 mg, 1.00 mmol) in 1 mL of a 1:1 isopropanol:water mixture was treated with 3 drops of Et_2NH and the mixture was stirred for 30 min at 80 °C. The mixture was removed from the heat and was treated with 4 mL of AcOH and 1 drop of concentrated HCl. The mixture was heated at 80 °C for 1 h. The mixture was removed from the heat and a solid crashed out of solution overnight. The solid was isolated by filtration and was dissolved in 5 mL EtOH and treated with hydrazine hydrate (70 mL) and the mixture was stirred at 80 °C for 1 h. The reaction mixture was removed from heat and 36C1 was isolated by vacuum filtration as a brown solid (72 mg, 22% yield).

Mp: 234-237 °C; ^1H (400 MHz, DMSO-d_6): δ 10.6 (bs, 1H), 7.96 (s, 1H), 7.76 (s, 1H), 7.41 (d, J = 7.2 Hz, 1H), 7.41 (s, 1H), 7.80 (d, J = 8.8 Hz, 1H), 7.67 (d, J = 3.2 Hz, 1H), 6.59 (t, J = 1.6 Hz, 1H), 3.35 (s, 2H); ^{13}C (75.5 MHz, DMSO-d_6): δ 177.9, 147.6, 143.7, 140.9, 139.9, 134.1, 131.9, 126.6, 113.6, 111.7, 109.9, 109.6, 68.8, 43.5; FTIR (neat, thin film): 3268 (m), 1709 (s), 1617 (m), 1474 (m) cm^{-1}; HRMS (ESI) calcd for $C_{14}H_{11}BrN_3O_2$ $[M+1]^+$: 332.0029; found: 332.0054.

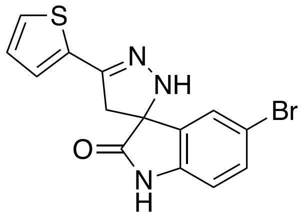

36C2

5-Bromo-5'-(thiophen-2-yl)-2',4'-dihydrospiro[indoline-3,3'-pyrazol]-2-one (36C2**)**

Following Procedure A, 2-acetylthiophene (1 mmol) was converted to 36C2 (107 mg, 31% yield). Mp: 230-235 °C; ^1H (400 MHz, DMSO-d_6): δ 10.5 (bs, 1H), 7.90 (s, 1H), 7.55 (d, J = 4.8 Hz, 1H), 7.42 (d, J = 7.6 Hz, 1H), 7.41 (s, 1H), 7.19 (d, J = 4.8 Hz, 1H), 7.09 (dd, J = 4.2, 3.6 Hz, 1H), 6.81 (d, J = 8.4 Hz, 1H), 3.43 (s, 2H); ^{13}C (75.5 MHz, DMSO-d_6): δ 177.9, 144.0, 140.9, 135.8, 134.1, 131.9, 127.6, 127.0, 126.6, 113.6, 111.7, 69.3, 44.2; FTIR (neat, thin film): 3272 (m), 1694 (m), 1725 (s), 1615 (m), 1474 (m) cm^{-1}; HRMS (ESI) calcd for $C_{14}H_{11}BrN_3OS$ $[M+1]^+$: 347.9801; found: 347.9829.

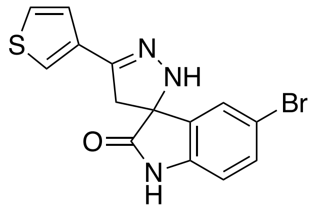

36C3

5-Bromo-5'-(thiophen-3-yl)-2',4'-dihydrospiro[indoline-3,3'-pyrazol]-2-one (36C3**)**
Following Procedure A, 36C3 was obtained from 3-acetylthiophene. Mp: 208-211 °C; ^{1}H (400 MHz, DMSO-d$_6$): δ 10.5 (bs, 1H), 7.80 (bs, 1H), 7.63-7.59 (m, 2H),7.44-7.35 (m, 3 H), 6.81 (d, J = 8.0 Hz, 1 H), 3.39 (s, 2H); ^{13}C (75.5 MHz, DMSO-d$_6$): δ 178.2, 144.8, 140.8, 134.8, 134.5, 131.7, 127.1, 126.5, 125.4, 123.8,113.6, 111.6, 68.9, 44.4; FTIR (neat, thin film): 3054.3, 1727.4, 1618.7 cm^{-1}; HRMS (ESI) calcd for $C_{14}H_{10}BrN_3NaOS$ [M+Na]$^+$: 369.9620; found: 369.9628.

Procedure B:
A mixture of 5-bromoisatin (226 mg, 1.00 mmol), 2-3 drops of diethylamine, 5 mL of ethanol (absolute), and the arylmethylketone (1.00 mmol) was refluxed for 30 minutes and then allowed to cool overnight. The mixture was then concentrated, providing the crude product as a brown solid. The crude product was dissolved in glacial acetic acid (1.6 mL) and was treated with 1 drop of concentrated HCl (1 drop). The resulting mixture was heated at 80 °C for 30-45 min. Upon cooling to rt, the solution was diluted with ethanol (absolute) and the mixture was filtered and the crude red solid was dried in vacuo. The crude product was dissolved in 5 mL of ethanol (absolute) and was treated with hydrazine hydrate (74 μL). The mixture was refluxed for 6 h, then was cooled to rt and was filtered. The crude solid was recrystallized from ethanol (absolute).

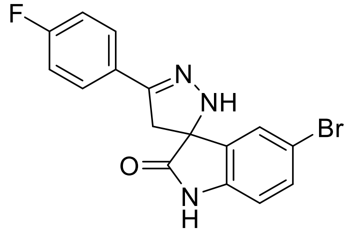

36C4

5-Bromo-5'-(4-fluorophenyl)-2',4'-dihydrospiro[indoline-3,3'-pyrazol]-2-one (36C4**)**
Following Procedure B, 36C4 was obtained as a greyish white solid in 12% yield (41.6 mg) from 4-fluoroacetophenone (138 mg, 1 mmol). Mp: 251-253 °C; ^{1}H NMR (400 HMz, DMSO-d$_6$): δ 10.53 (s, 1H), 7.96 (s, 1H), 7.69 (q, J = 4 Hz, 2H), 7.42 (apparent d, J = 8 Hz, 2H), 7.26 (t, J = 8 Hz, 2H), 6.82 (d, J = 8 Hz, 1H), 3.44 (s, 2H); ^{13}C NMR (125 MHz, DMSO-d$_6$): δ 178.6, 163.9, 161.4, 147.3, 141.3, 134.9, 132.3, 129.6, 129.6, 128.4, 128.3, 127.1, 116.1, 115.9, 114.1, 112.1, 69.8, 44.1; FTIR (neat, thin film): 3332, 3289, 3166, 3050, 1702, 1221, 821.6, 541.3; HRMS (ESI) calculated for $C_{16}H_{11}BrFN_3O$ [M+Na]$^+$: 381.9967, found 381.9960.

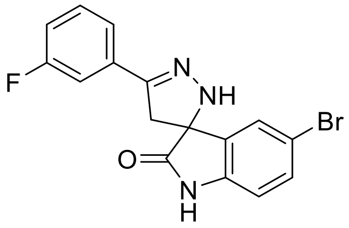

36C5

5-Bromo-5'-(3-fluorophenyl)-2',4'-dihydrospiro[indoline-3,3'-pyrazol]-2-one (36C5**)**
Using Procedure B, 36C5 was obtained as a greyish white solid in 17% yield (61.2 mg) from 3-fluoroacetophenone (138 mg, 1 mmol). Mp: 253-255 °C; ^{1}H NMR (400 MHz, DMSO-d$_6$): δ 10.55 (s, 1H), 8.13 (s,1H), 7.41-7.49 (m, 5H), 7.17-7.21 (m, 1H), 6.83 (d, J = 8 Hz, 1H), 3.45 (s, 2H); ^{13}C NMR (125 MHz, DMSO-d$_6$): δ 178.5, 164.0, 161.6, 146.9, 141.3, 135.4, 134.8, 132.3, 131.1, 127.2, 122.3, 115.5, 114.2, 112.5, 69.9, 43.8; FTIR (neat, thin film): 3426, 3266, 3192, 3110, 1714, 851.8 cm^{-1}; HRMS (ESI) calculated for $C_{16}H_{11}BrFN_3O$ [M+Na]$^+$: 381.9967, found 381.9964.

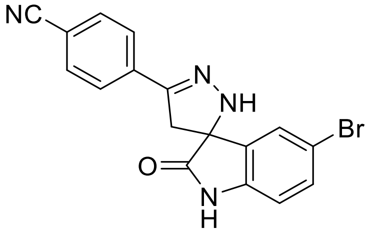

36C6

4-(5-Bromo-2-oxo-2',4'-dihydrospiro[indoline-3,3'-pyrazol]-5'-yl) benzonitrile (36C6**)**
Using Procedure B, 36C6 was obtained as a brown solid in 5% yield (17.0 mg) from 4-acetylbenzonitrile (145 mg, 1 mmol). Mp >260 °C; ^{1}H NMR (400 MHz, DMSO-d$_6$): δ 10.57 (s, 1H), 8.41 (s, 1H), 7.82 (dd, J = 8, 24 Hz, 4H), 7.44 (d, J = 8 Hz, 2H), 6.83 (d, J = 8 Hz, 1H), 3.48 (s, 2H); ^{13}C NMR (125 MHz, DMSO-d$_6$): δ 178.3, 146.1, 141.4, 137.4, 134.5, 133.0, 133.0, 132.5, 127.3, 126.6, 126.6, 119.4, 114.2, 112.2, 110.5, 70.1, 43.2; FTIR (neat, thin film): 3328, 3177, 3118, 2228, 1705, 1476, 559.9 cm^{-1}; HRMS (ESI) calculated for $C_{17}H_{11}BrN_4O$ [M+Na]$^+$: 389.0014, found 389.0010.

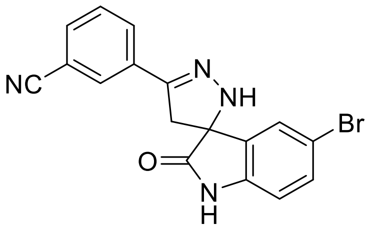

36C7

3-(5-Bromo-2-oxo-2',4'-dihydrospiro[indoline-3,3'-pyrazol]-5'-yl) benzonitrile (36C7**)**

Using Procedure B, 36C7 was obtained as a greyish white solid in 30% yield (109.4 mg) from 3-acetylbenzonitrile (145 mg, 1 mmol). Mp >260 °C; ^{1}H NMR (400 MHz, DMSO-d$_6$): δ 10.57 (s, 1H), 8.25 (s, 1H), 7.99 (apparent d, J = 8 Hz, 2H), 7.81 (d, J = 8 Hz, 1H), 7.63 (t, J = 8 Hz, 1H), 7.42-7.45 (m, 2H), 6.83 (d, J = 8 Hz, 1H), 3.49 (s, 2H); ^{13}C NMR (125 MHz, DMSO-d$_6$): δ 178.4, 146.1, 141.3, 134.7, 134.2, 132.4, 132.1, 130.4, 130.3, 129.5, 127.2, 119.1, 114.2, 112.3, 112.2, 69.9, 43.5; FTIR (neat, thin film): 3421, 3255, 3111, 3069, 2235, 1712, 1213, 679.2, 539.5 cm^{-1}: HRMS (ESI) calculated for $C_{17}H_{11}BrN_4O$ [M+Na]$^+$: 389.0014, found 389.0007.

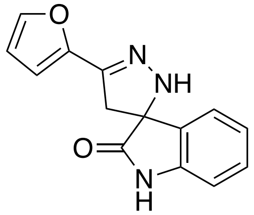

31C1

5'-(Furan-2-yl)-2',4'-dihydrospiro[indoline-3,3'-pyrazol]-2-one (31C1**)**

Following Procedure A, 31C1 was obtained from 2-acetylfuran. Mp: 192-196 °C; ^{1}H NMR (400 MHz, DMSO-d$_6$) δ 10.35 (s, 1H), 7.88 (s, 1H), 7.72 (s, 1H), 7.24-7.17 (m, 2H), 7.94 (t, J = 7.2 Hz,1H), 6.80 (d, J = 7.4 Hz, 1H), 6.56 (s, 1H), 6.55 (s, 1H), 3.33 (d, J = 17 Hz, 1H), 3.22 (d, J = 17 Hz, 1H); ^{13}C NMR (75.5 MHz, DMSO-d$_6$) δ 178.8, 154.0, 151.3, 148.2, 144.1, 142.0, 140.0, 132.2, 129.6, 124.1, 122.6, 112.1, 110.1, 69.2, 44.1; FTIR (neat, thin film) 3418.9 (br), 3266.5 (m), 1688.6 (s), 1471.1 (m) cm^{-1}; HRMS (ESI) calcd for $C_{14}H_{11}N_3NaO_2$ [M+Na]$^+$: 276.0743; found: 276.0742.

References

(1) Liu, H.-Q.; Wang, D.-C.; Wu, F.; Tang, W.; Ouyang, P.-K., Synthesis and biological evaluation of 5'-phenyl-3'H-spiro-[indoline-3,2'-[1,3,4]oxadiazol]-2-one analogs. *Chin. Chem. Lett.* **2013,** *24*, 929-933.
(2) Gangarapu, K.; Thumma, G.; Manda, S.; Jallapally, A.; Jarapula, R.; Rekulapally, S., Design, synthesis and molecular docking of novel structural hybrids of substituted isatin based pyrazoline and thiadiazoline as antitumor agents. *Med. Chem. Res.* **2017,** *26* (4), 819-829.
(3) Azizian, J.; Shaabanzadeh, M.; Hatamjafari, F.; Mahammadizadeh, M. R., One-pot rapid and efficient synthesis of new spiro derivatives of 11H-indeno[1,2-b]quinoxalin-11-one, 6H-indeno[1,2-b]pyrido[3,2-e]pyrazin-6-one and isatin-based 2-pyrazolines. *Arkivoc* **2006,** *11*, 47-58.
(4) Falconer, S. B.; Reid-Yu, S. A.; King, A. M.; Gehrke, S. S.; Wang, W.; Britten, J. F.; Coombes, B. K.; Wright, G. D.; Brown, E. D., Zinc Chelation by a Small-Molecule Adjuvant Potentiates Meropenem Activity in Vivo against NDM-1-Producing Klebsiella pneumoiae. *ACS Infect. Dis.* **2015,** *1*, 533-543.
(5) Youssef, M. S. K.; Abeed, A. A. O., Synthesis and antimicrobial activity of some novel 2-thienyl substituted heterocycles. *Heterocycl. Commun.* **2014,** *20* (1), 25-31.

(6) Pauli, G. F.; Chen, S. N.; Simmler, C.; Lankin, D. C.; Godecke, T.; Jaki, B. U.; Friesen, J. B.; McAlpine, J. B.; Napolitano, J. G., Importance of Purity Evanluation and the Potential of Quantitative 1H NMR as a Purity Assay. *J. Med. Chem.* **2014**, *57*, 9220-9231.

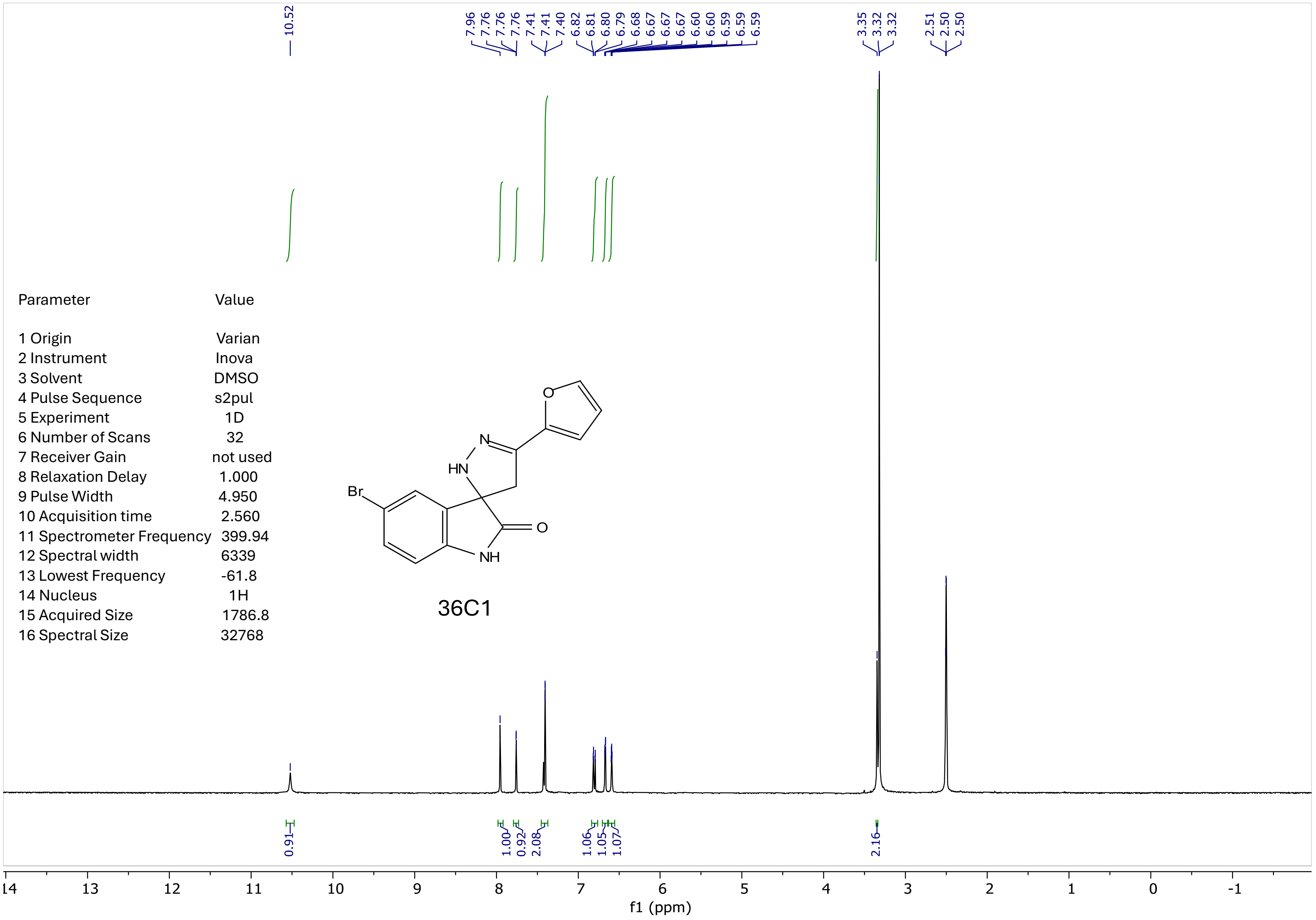

Parameter	Value
1 Origin	Varian
2 Instrument	Inova
3 Solvent	DMSO
4 Pulse Sequence	s2pul
5 Experiment	1D
6 Number of Scans	32
7 Receiver Gain	not used
8 Relaxation Delay	1.000
9 Pulse Width	4.950
10 Acquisition time	2.560
11 Spectrometer Frequency	399.94
12 Spectral width	6339
13 Lowest Frequency	-61.8
14 Nucleus	1H
15 Acquired Size	1786.8
16 Spectral Size	32768

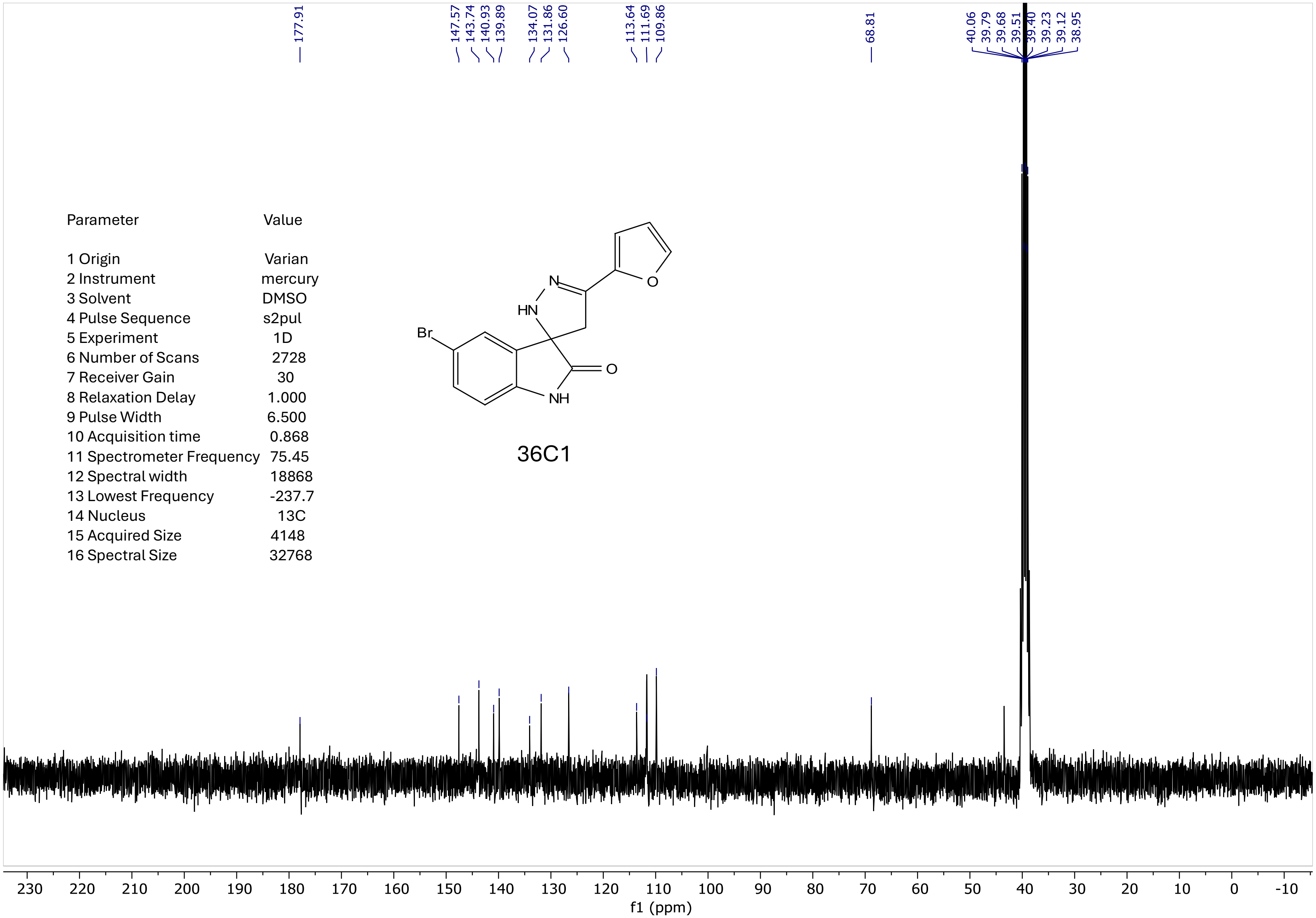
177.91
147.57
143.74
140.93
139.89
134.07
131.86
126.60
113.64
111.69
109.86
68.81
40.06
39.79
39.68
39.51
39.40
39.23
39.12
38.95
Parameter Value
1 Origin Varian
2 Instrument mercury
3 Solvent DMSO
4 Pulse Sequence s2pul
5 Experiment 1D
6 Number of Scans 2728
7 Receiver Gain 30
8 Relaxation Delay 1.000
9 Pulse Width 6.500
10 Acquisition time 0.868
11 Spectrometer Frequency 75.45
12 Spectral width 18868
13 Lowest Frequency -237.7
14 Nucleus 13C
15 Acquired Size 4148
16 Spectral Size 32768
Br
HN
N
O
O
NH
36C1
230 220 210 200 190 180 170 160 150 140 130 120 110 100 90 80 70 60 50 40 30 20 10 0 -10
f1 (ppm)

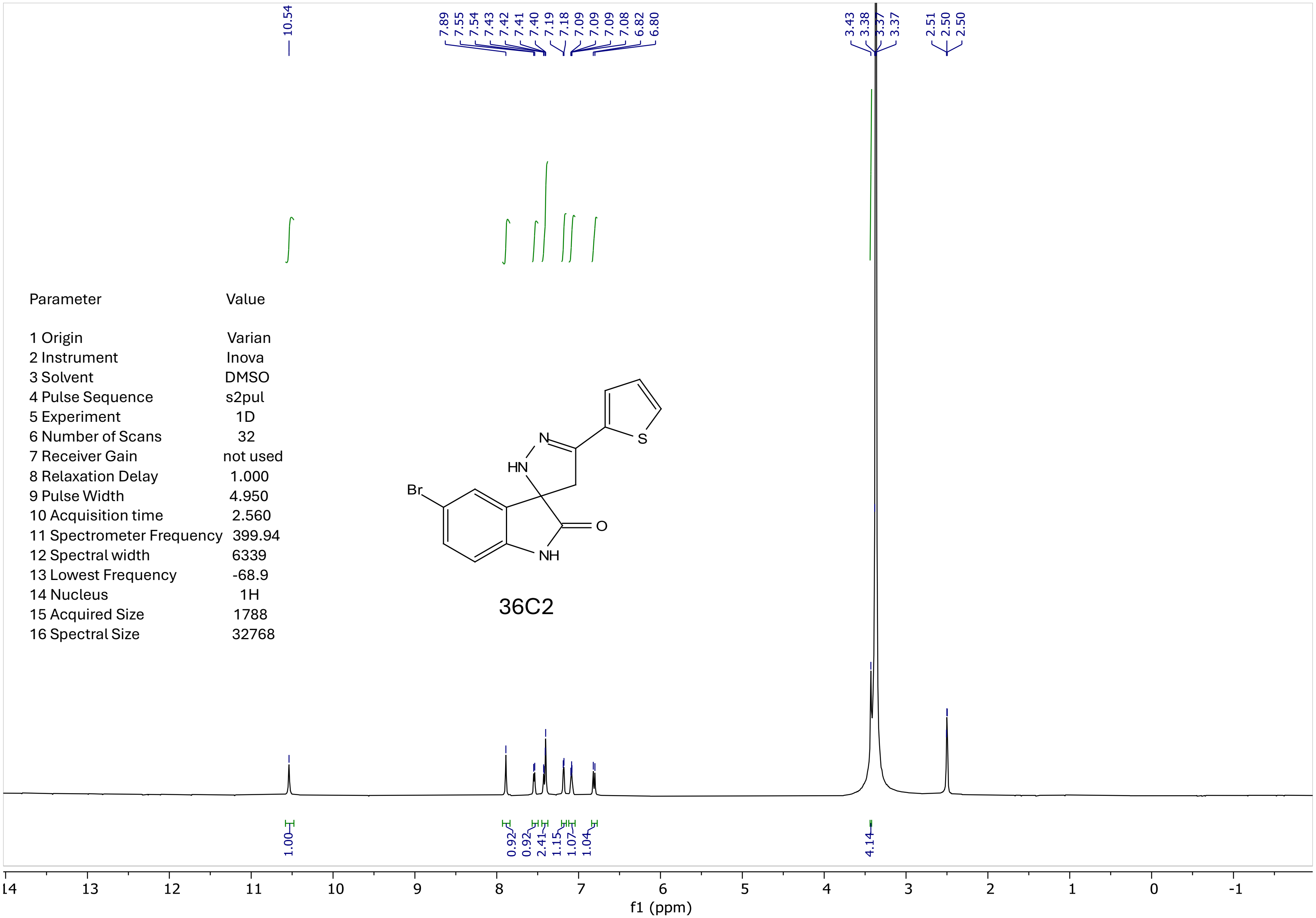
10.54
7.89
7.55
7.54
7.43
7.42
7.41
7.40
7.19
7.18
7.09
7.09
7.09
7.08
6.82
6.80
3.43
3.38
3.37
3.37
2.51
2.50
2.50
Parameter Value
1 Origin Varian
2 Instrument Inova
3 Solvent DMSO
4 Pulse Sequence s2pul
5 Experiment 1D
6 Number of Scans 32
7 Receiver Gain not used
8 Relaxation Delay 1.000
9 Pulse Width 4.950
10 Acquisition time 2.560
11 Spectrometer Frequency 399.94
12 Spectral width 6339
13 Lowest Frequency -68.9
14 Nucleus 1H
15 Acquired Size 1788
16 Spectral Size 32768
Br
HN
N
S
O
NH
36C2
1.00
0.92
0.92
2.41
1.15
1.07
1.04
4.14
14
13
12
11
10
9
8
7
6
5
4
3
2
1
0
-1
f1 (ppm)

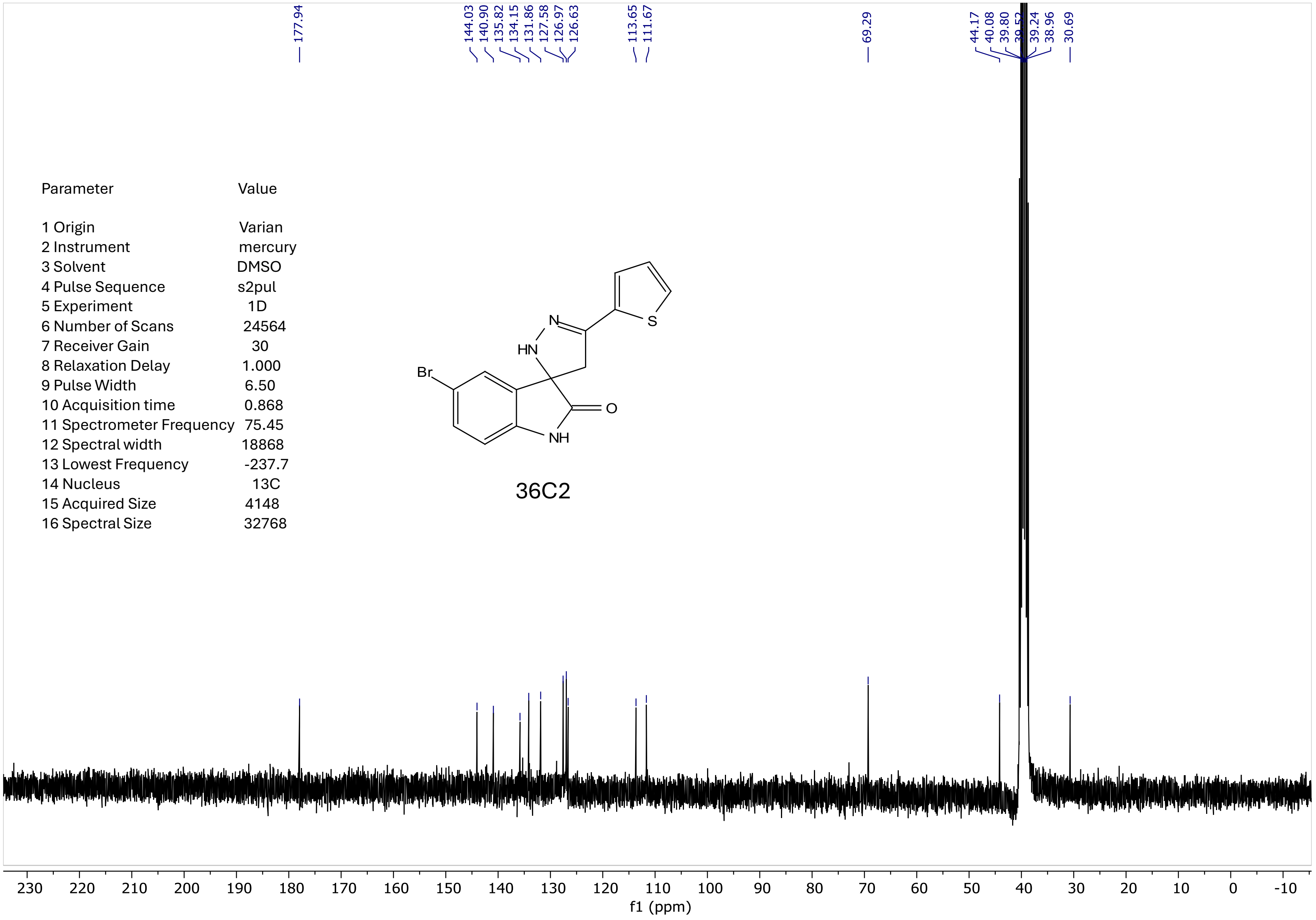

36C2

Parameter	Value
1 Origin	Varian
2 Instrument	mercury
3 Solvent	DMSO
4 Pulse Sequence	s2pul
5 Experiment	1D
6 Number of Scans	24564
7 Receiver Gain	30
8 Relaxation Delay	1.000
9 Pulse Width	6.50
10 Acquisition time	0.868
11 Spectrometer Frequency	75.45
12 Spectral width	18868
13 Lowest Frequency	-237.7
14 Nucleus	13C
15 Acquired Size	4148
16 Spectral Size	32768

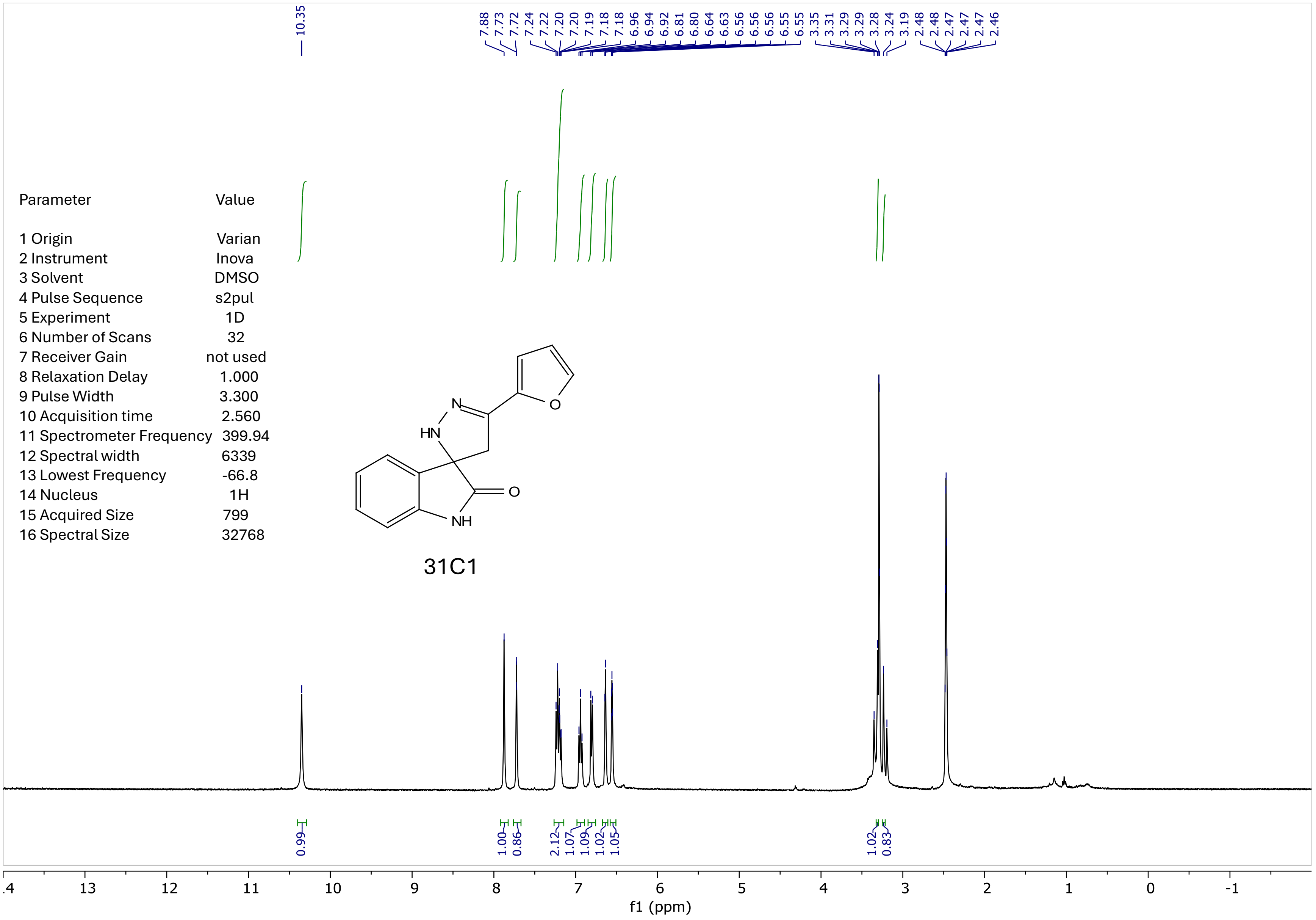
10.35
7.88 7.73 7.72 7.24 7.22 7.20 7.20 7.19 7.18 7.18 6.96 6.94 6.92 6.81 6.80 6.64 6.63 6.56 6.56 6.56 6.55 6.55 3.35 3.31 3.29 3.29 3.28 3.24 3.19 2.48 2.48 2.47 2.47 2.47 2.46
Parameter Value
1 Origin Varian
2 Instrument Inova
3 Solvent DMSO
4 Pulse Sequence s2pul
5 Experiment 1D
6 Number of Scans 32
7 Receiver Gain not used
8 Relaxation Delay 1.000
9 Pulse Width 3.300
10 Acquisition time 2.560
11 Spectrometer Frequency 399.94
12 Spectral width 6339
13 Lowest Frequency -66.8
14 Nucleus 1H
15 Acquired Size 799
16 Spectral Size 32768
N
HN
O
O
NH
31C1
0.99
1.00
0.86
2.12
1.07
1.09
1.02
1.05
1.02
0.83
.4 13 12 11 10 9 8 7 6 5 4 3 2 1 0 -1
f1 (ppm)

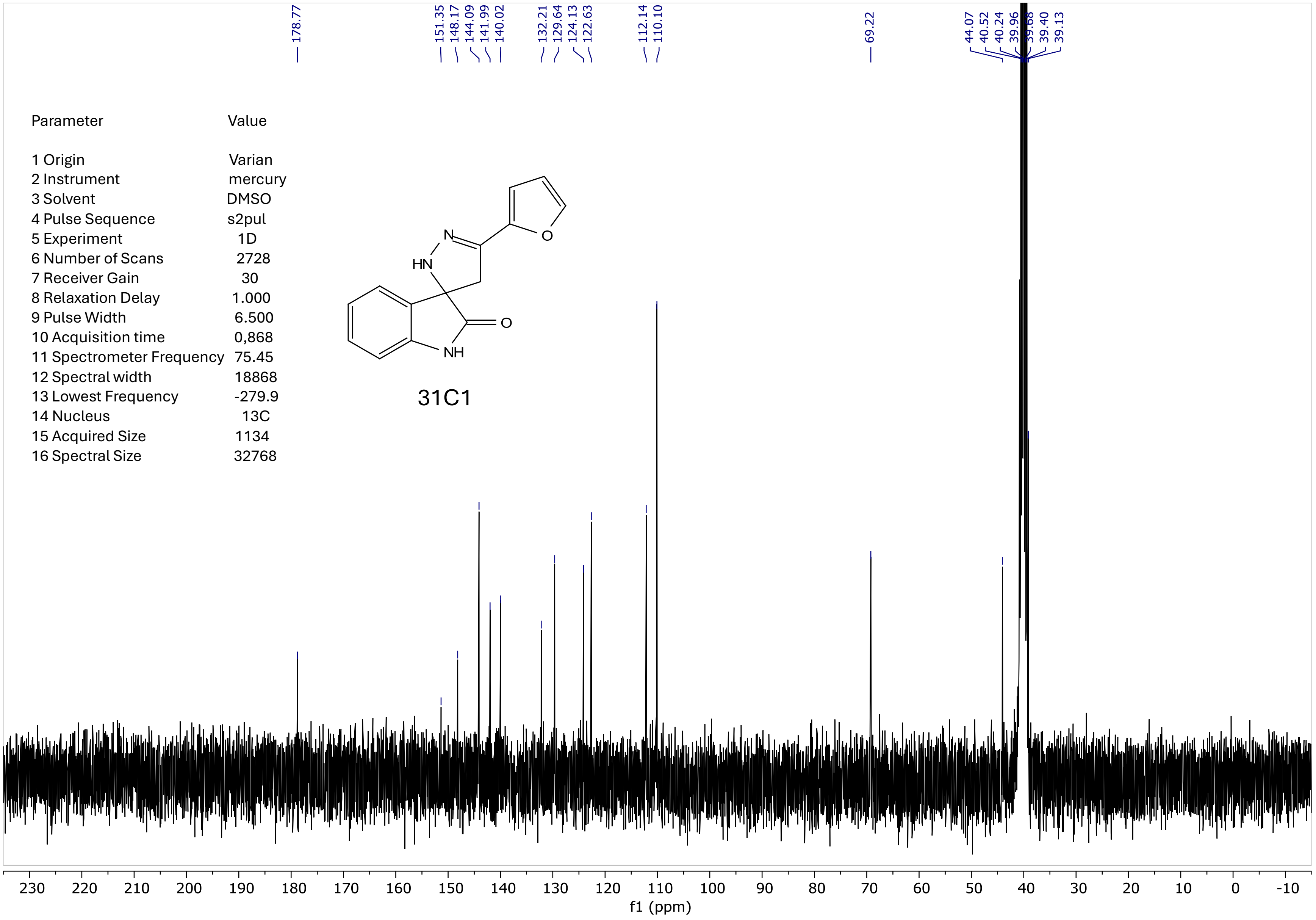

Parameter	Value
1 Origin	Varian
2 Instrument	mercury
3 Solvent	DMSO
4 Pulse Sequence	s2pul
5 Experiment	1D
6 Number of Scans	2728
7 Receiver Gain	30
8 Relaxation Delay	1.000
9 Pulse Width	6.500
10 Acquisition time	0,868
11 Spectrometer Frequency	75.45
12 Spectral width	18868
13 Lowest Frequency	-279.9
14 Nucleus	13C
15 Acquired Size	1134
16 Spectral Size	32768

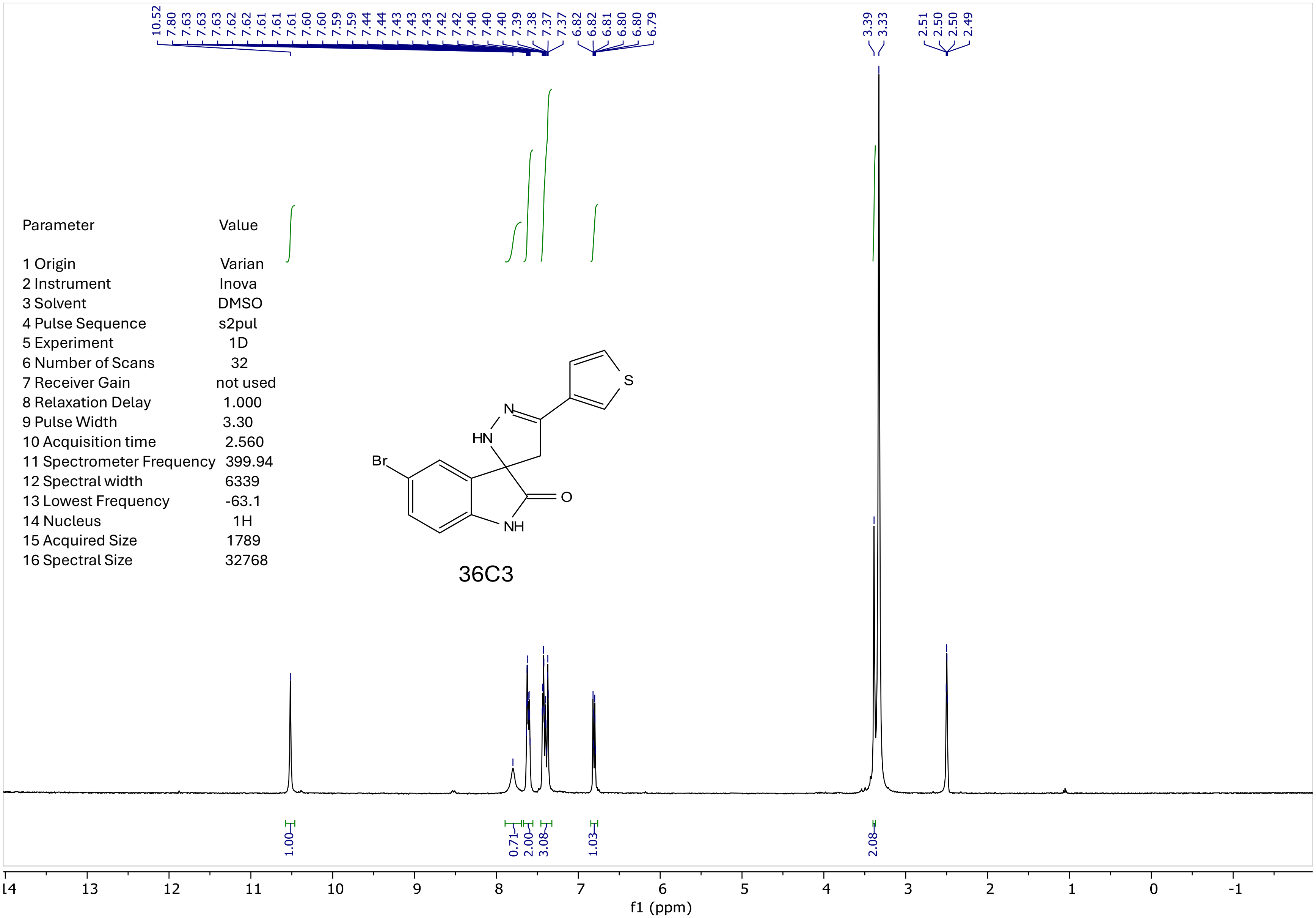

Parameter	Value
1 Origin	Varian
2 Instrument	Inova
3 Solvent	DMSO
4 Pulse Sequence	s2pul
5 Experiment	1D
6 Number of Scans	32
7 Receiver Gain	not used
8 Relaxation Delay	1.000
9 Pulse Width	3.30
10 Acquisition time	2.560
11 Spectrometer Frequency	399.94
12 Spectral width	6339
13 Lowest Frequency	-63.1
14 Nucleus	1H
15 Acquired Size	1789
16 Spectral Size	32768

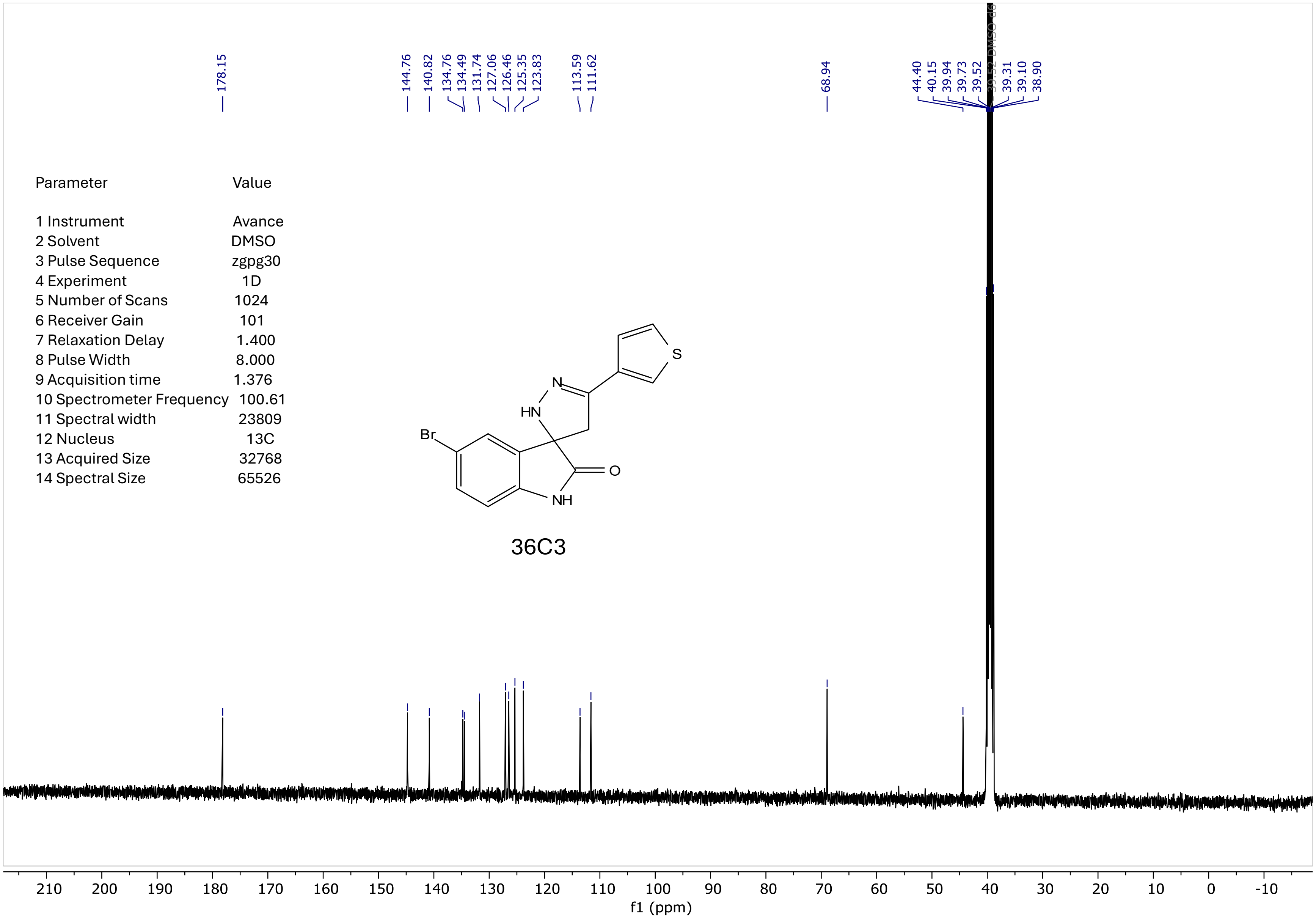

Parameter	Value
1 Instrument	Avance
2 Solvent	DMSO
3 Pulse Sequence	zgpg30
4 Experiment	1D
5 Number of Scans	1024
6 Receiver Gain	101
7 Relaxation Delay	1.400
8 Pulse Width	8.000
9 Acquisition time	1.376
10 Spectrometer Frequency	100.61
11 Spectral width	23809
12 Nucleus	13C
13 Acquired Size	32768
14 Spectral Size	65526

36C3

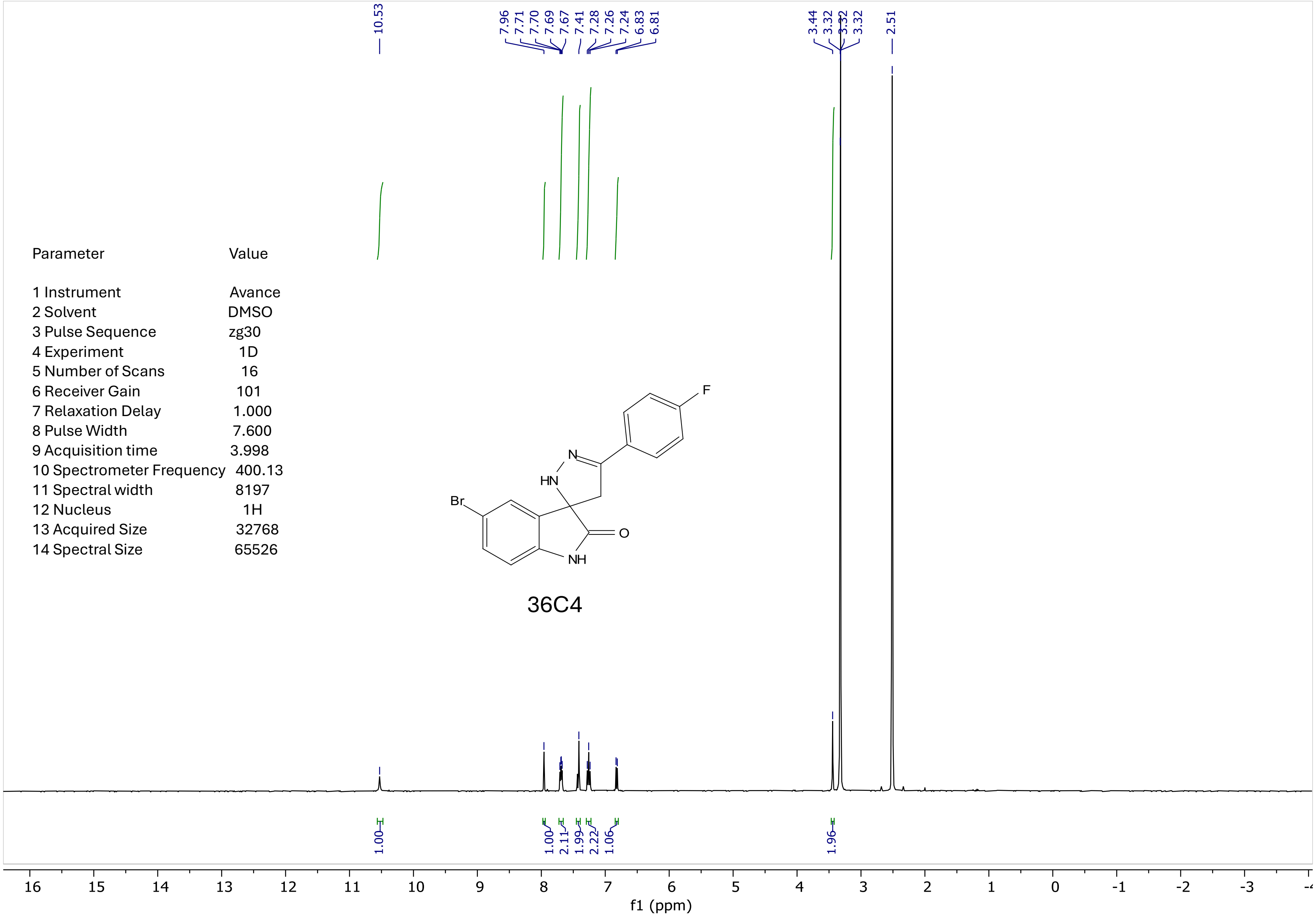

Parameter	Value
1 Instrument	Avance
2 Solvent	DMSO
3 Pulse Sequence	zg30
4 Experiment	1D
5 Number of Scans	16
6 Receiver Gain	101
7 Relaxation Delay	1.000
8 Pulse Width	7.600
9 Acquisition time	3.998
10 Spectrometer Frequency	400.13
11 Spectral width	8197
12 Nucleus	1H
13 Acquired Size	32768
14 Spectral Size	65526

36C4

Parameter	Value
1 Instrument	Avance
2 Solvent	DMSO
3 Pulse Sequence	zgpg30
4 Experiment	1D
5 Number of Scans	1024
6 Receiver Gain	101
7 Relaxation Delay	1.400
8 Pulse Width	8.000
9 Acquisition time	1.376
10 Spectrometer Frequency	100.61
11 Spectral width	23809
12 Nucleus	13C
13 Acquired Size	32768
14 Spectral Size	65526

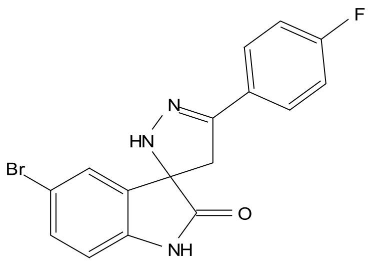

36C4

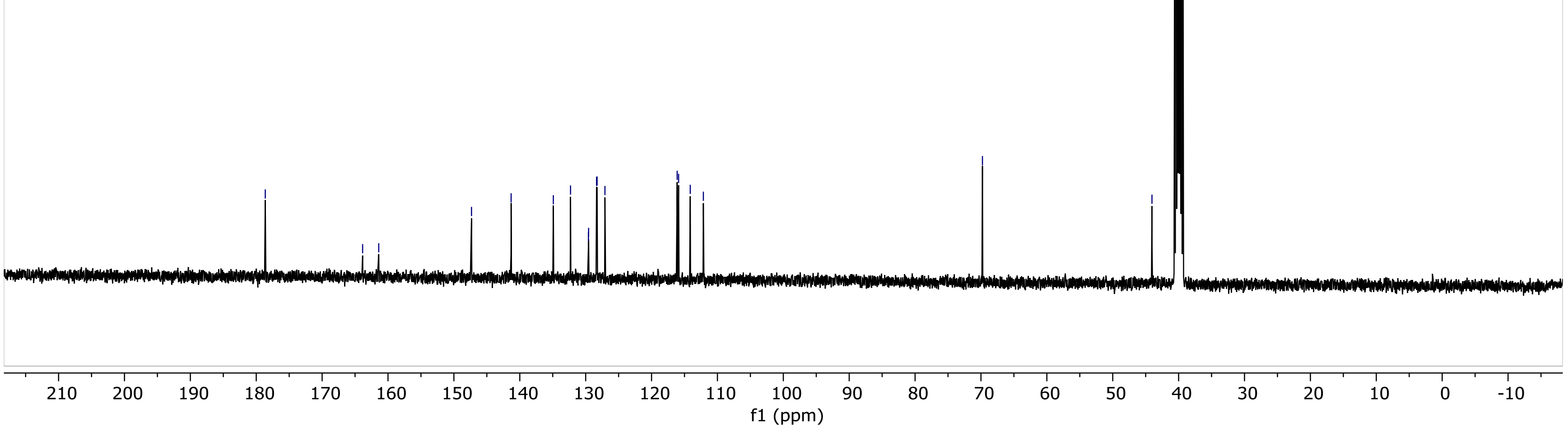

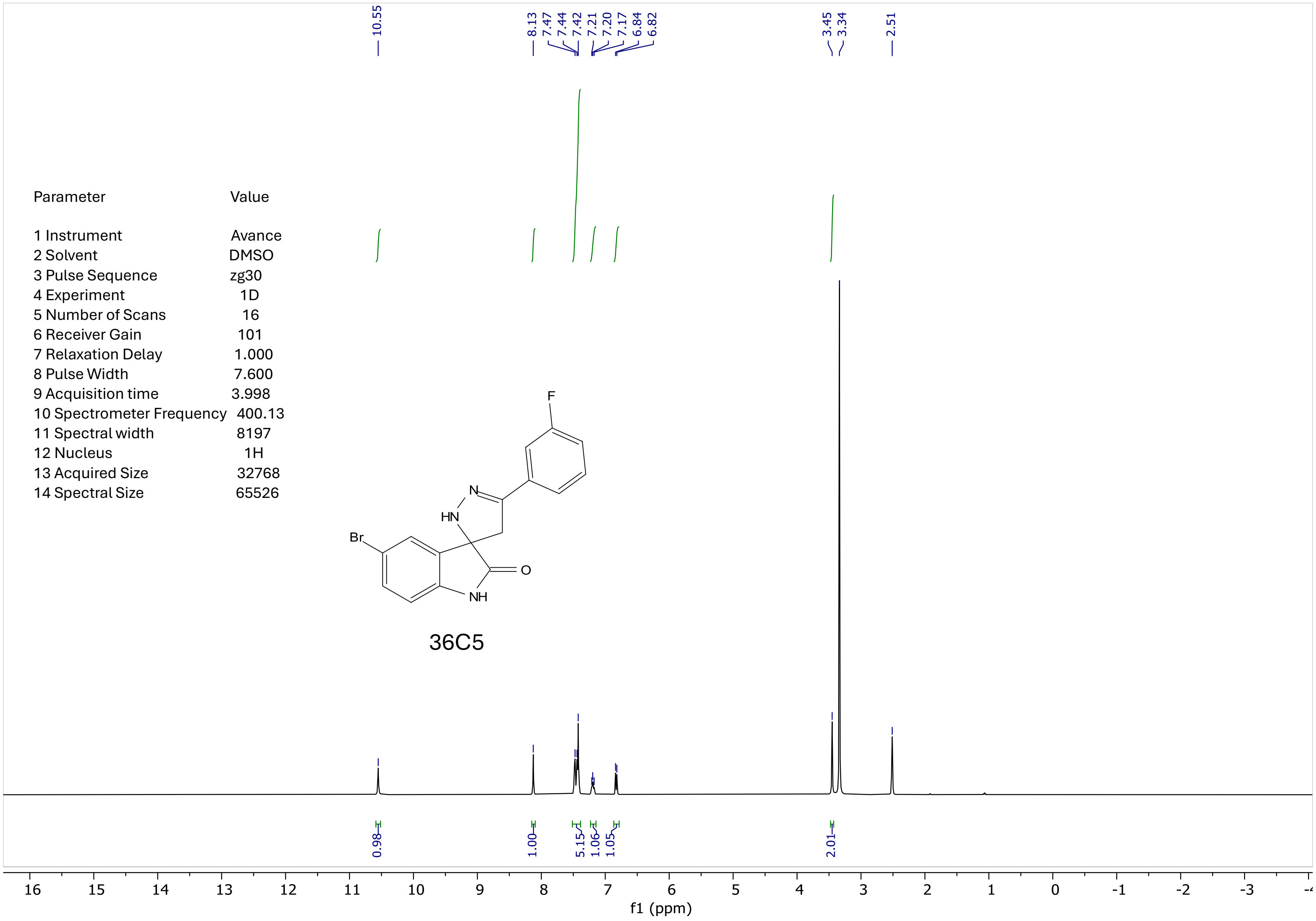

Parameter	Value
1 Instrument	Avance
2 Solvent	DMSO
3 Pulse Sequence	zg30
4 Experiment	1D
5 Number of Scans	16
6 Receiver Gain	101
7 Relaxation Delay	1.000
8 Pulse Width	7.600
9 Acquisition time	3.998
10 Spectrometer Frequency	400.13
11 Spectral width	8197
12 Nucleus	1H
13 Acquired Size	32768
14 Spectral Size	65526

36C5

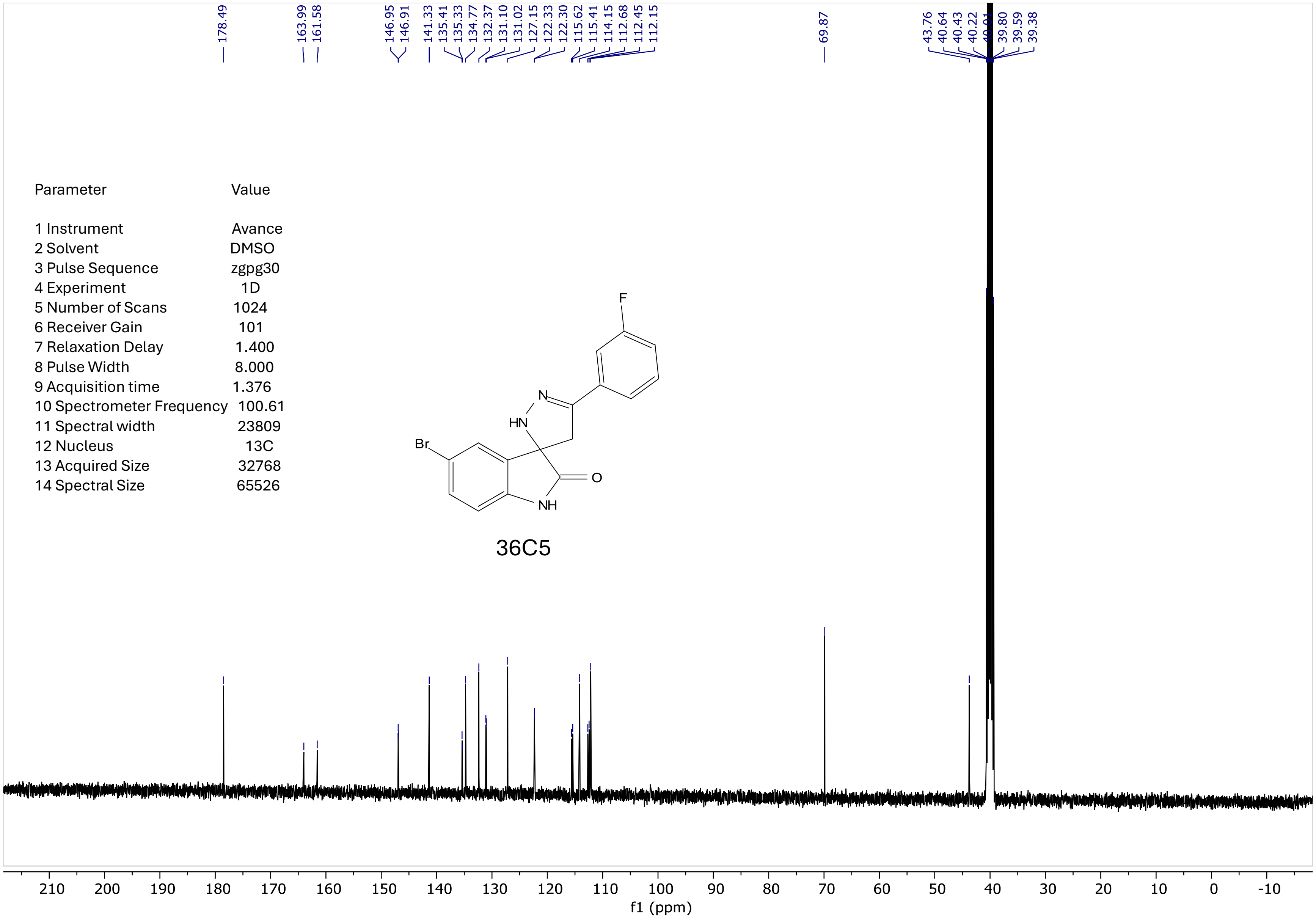

Parameter	Value
1 Instrument	Avance
2 Solvent	DMSO
3 Pulse Sequence	zgpg30
4 Experiment	1D
5 Number of Scans	1024
6 Receiver Gain	101
7 Relaxation Delay	1.400
8 Pulse Width	8.000
9 Acquisition time	1.376
10 Spectrometer Frequency	100.61
11 Spectral width	23809
12 Nucleus	13C
13 Acquired Size	32768
14 Spectral Size	65526

36C5

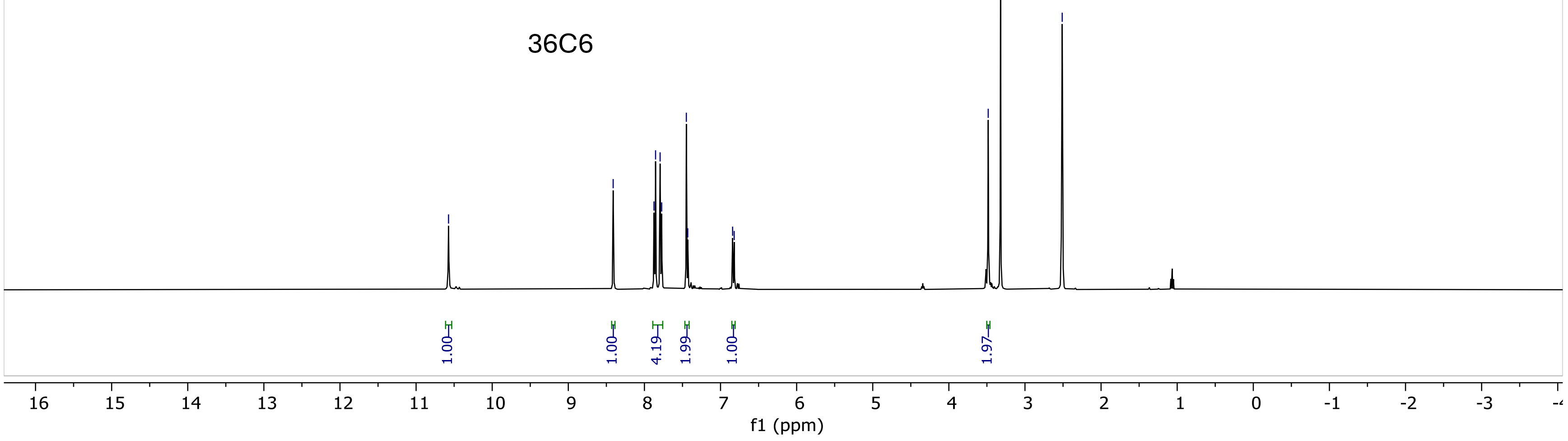

Parameter	Value
1 Instrument	Avance
2 Solvent	DMSO
3 Pulse Sequence	zg30
4 Experiment	1D
5 Number of Scans	16
6 Receiver Gain	101
7 Relaxation Delay	1.000
8 Pulse Width	7.600
9 Acquisition time	3.998
10 Spectrometer Frequency	400.13
11 Spectral width	8197
12 Nucleus	1H
13 Acquired Size	32768
14 Spectral Size	65526

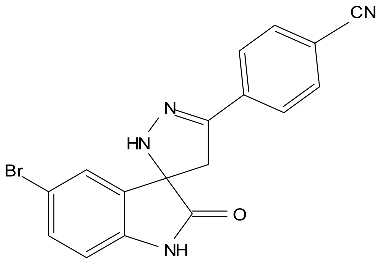

36C6

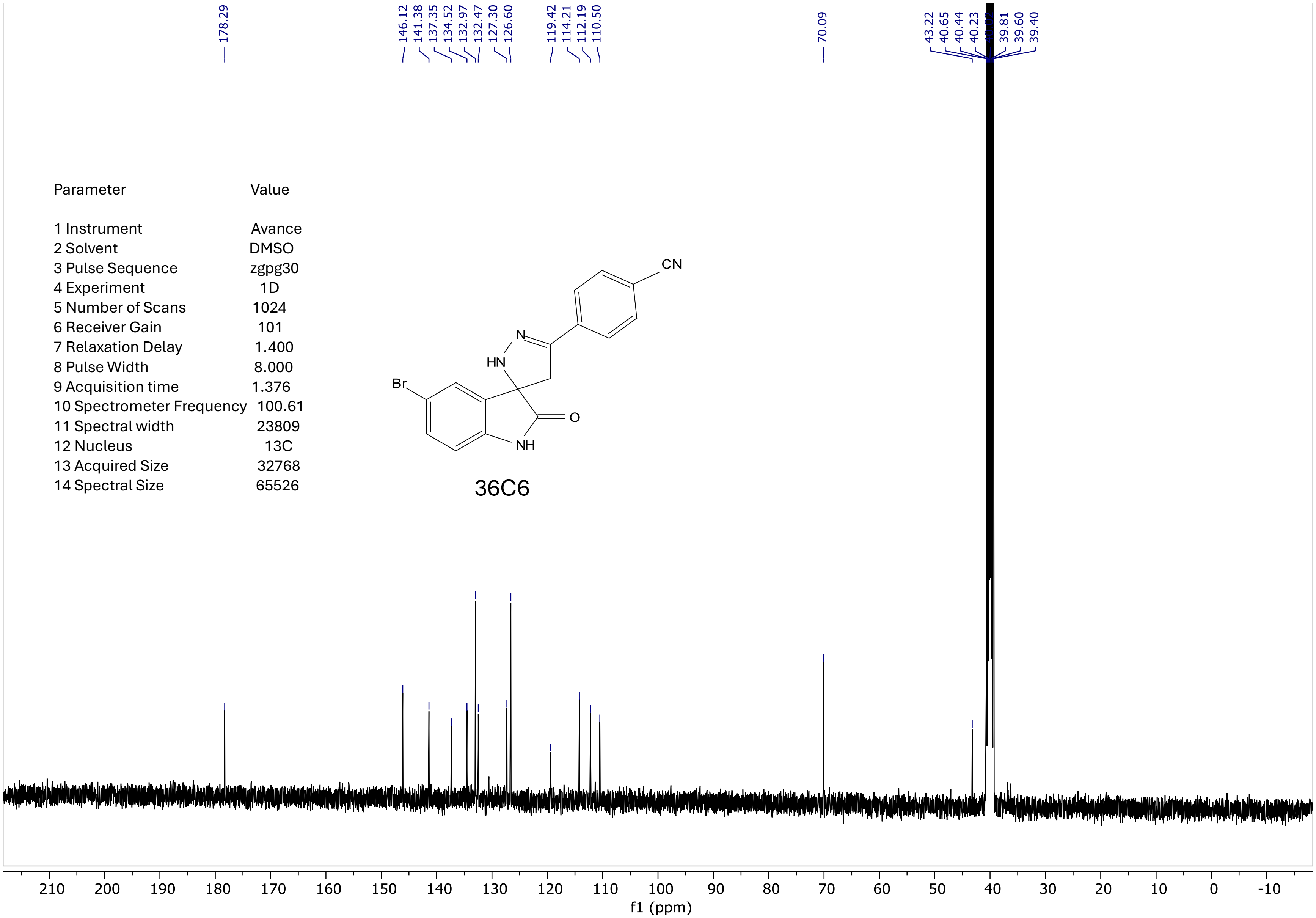

36C6

Parameter	Value
1 Instrument	Avance
2 Solvent	DMSO
3 Pulse Sequence	zgpg30
4 Experiment	1D
5 Number of Scans	1024
6 Receiver Gain	101
7 Relaxation Delay	1.400
8 Pulse Width	8.000
9 Acquisition time	1.376
10 Spectrometer Frequency	100.61
11 Spectral width	23809
12 Nucleus	13C
13 Acquired Size	32768
14 Spectral Size	65526

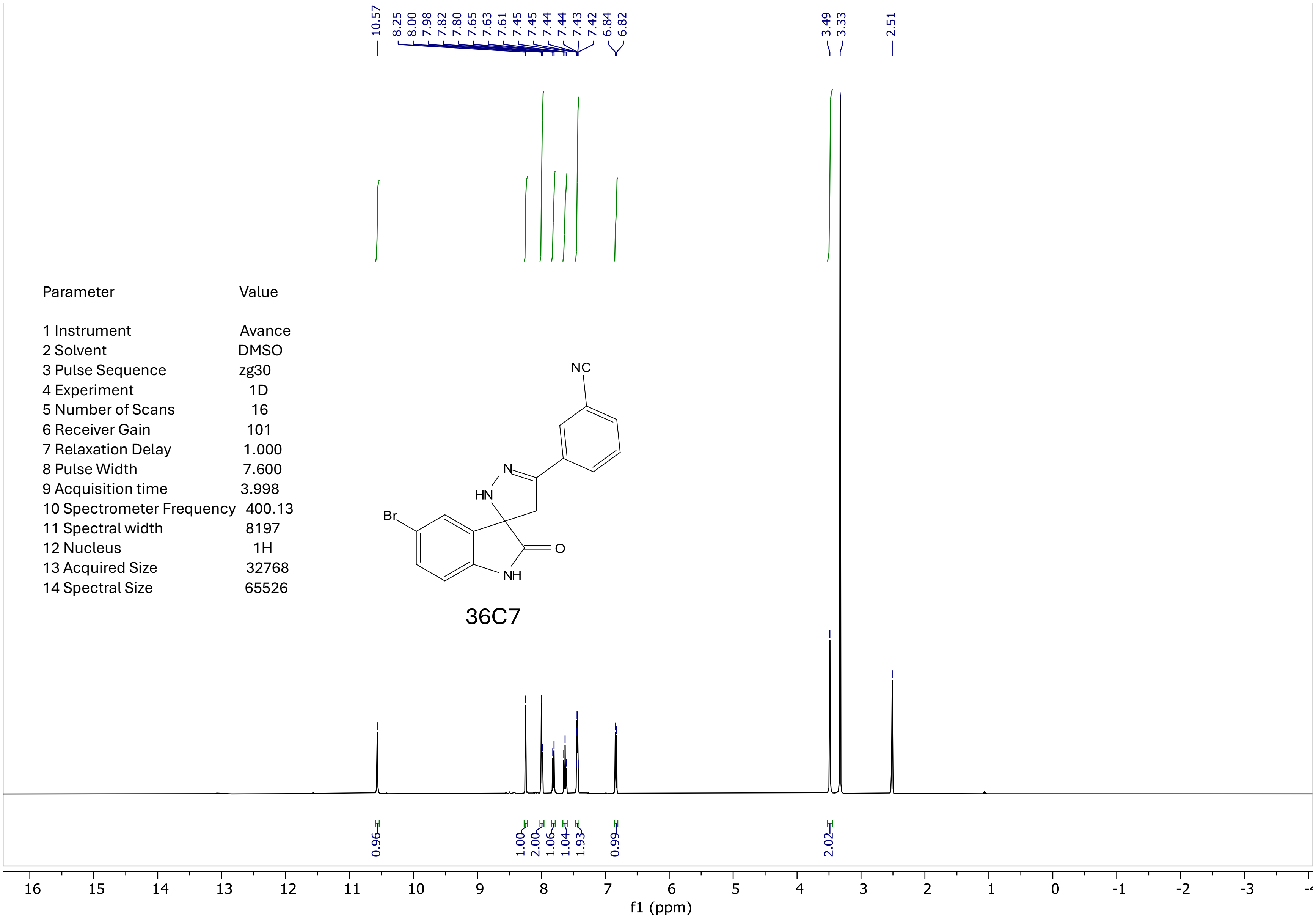

Parameter	Value
1 Instrument	Avance
2 Solvent	DMSO
3 Pulse Sequence	zg30
4 Experiment	1D
5 Number of Scans	16
6 Receiver Gain	101
7 Relaxation Delay	1.000
8 Pulse Width	7.600
9 Acquisition time	3.998
10 Spectrometer Frequency	400.13
11 Spectral width	8197
12 Nucleus	1H
13 Acquired Size	32768
14 Spectral Size	65526

36C7

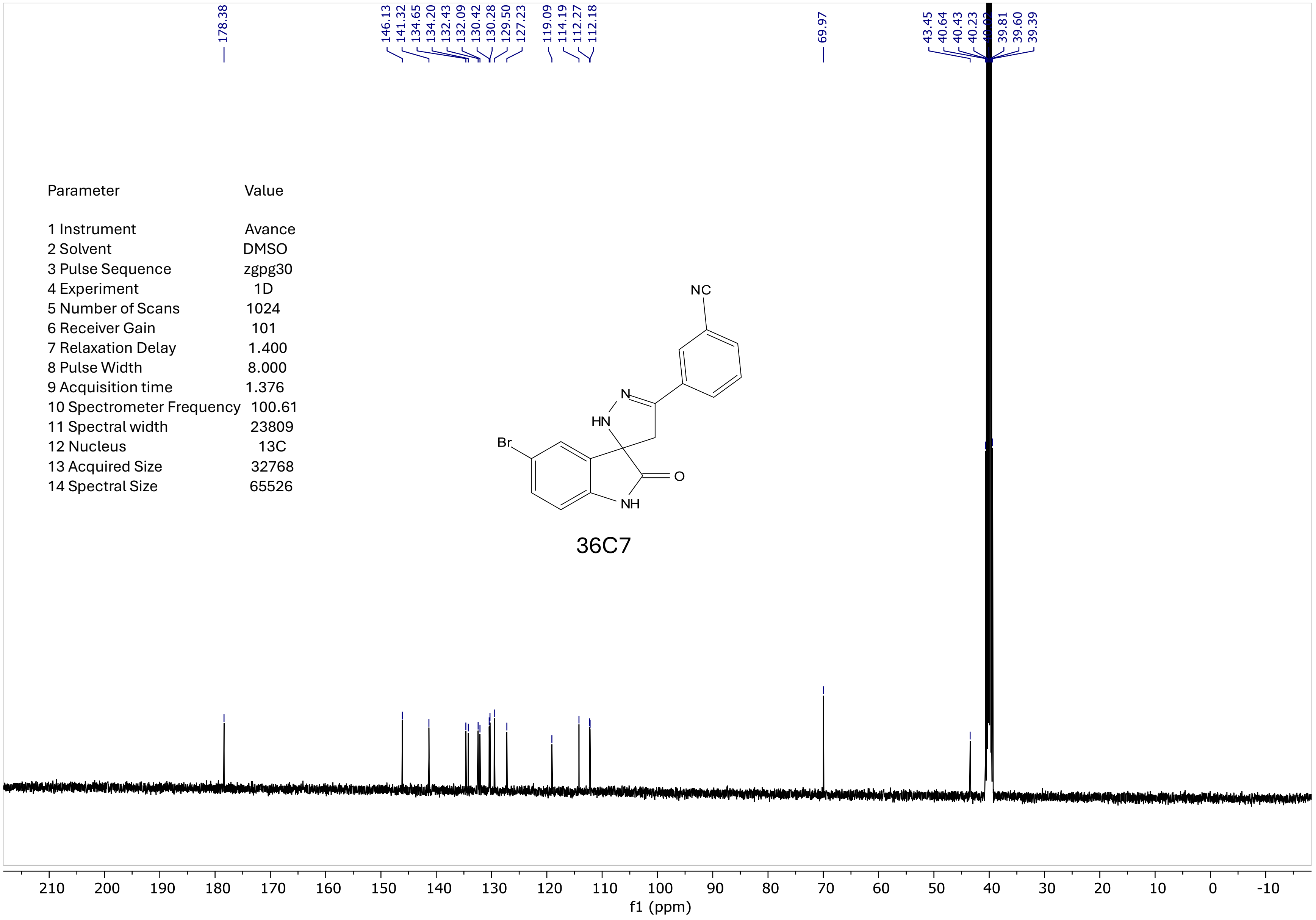

36C7

Parameter	Value
1 Instrument	Avance
2 Solvent	DMSO
3 Pulse Sequence	zgpg30
4 Experiment	1D
5 Number of Scans	1024
6 Receiver Gain	101
7 Relaxation Delay	1.400
8 Pulse Width	8.000
9 Acquisition time	1.376
10 Spectrometer Frequency	100.61
11 Spectral width	23809
12 Nucleus	13C
13 Acquired Size	32768
14 Spectral Size	65526
